# Two-step regulation by matrix Gla protein in brown adipose cell differentiation

**DOI:** 10.1016/j.molmet.2024.101870

**Published:** 2024-01-04

**Authors:** Li Zhang, Xinjiang Cai, Feiyang Ma, Xiaojing Qiao, Jaden Ji, Jocelyn A. Ma, Laurent Vergnes, Yan Zhao, Yucheng Yao, Xiuju Wu, Kristina I. Boström

**Affiliations:** 1 Division of Cardiology, David Geffen School of Medicine at UCLA, USA; 2 Molecular Biology Institute, University of California Los Angeles, Los Angeles, CA, USA; 3 Human Genetics, David Geffen School of Medicine at UCLA, Los Angeles, CA, USA

**Keywords:** Brown adipogenic differentiation, Matrix Gla protein (MGP), Bone morphogenetic protein signaling, Gene deletion, Single-cell sequencing analysis

## Abstract

**Objective:**

Bone morphogenetic protein (BMP) signaling is intricately involved in adipose tissue development. BMP7 together with BMP4 have been implicated in brown adipocyte differentiation but their roles during development remains poorly specified. Matrix Gla protein (MGP) inhibits BMP4 and BMP7 and is expressed in endothelial and progenitor cells. The objective was to determine the role of MGP in brown adipose tissue (BAT) development.

**Methods:**

The approach included global and cell-specific Mgp gene deletion in combination with RNA analysis, immunostaining, thermogenic activity, and *in vitro* studies.

**Results:**

The results revealed that MGP directs brown adipogenesis at two essential steps. Endothelial-derived MGP limits triggering of white adipogenic differentiation in the perivascular region, whereas MGP derived from adipose cells supports the transition of CD142-expressing progenitor cells to brown adipogenic maturity. Both steps were important to optimize the thermogenic function of BAT. Furthermore, MGP derived from both sources impacted vascular growth. Reduction of MGP in either endothelial or adipose cells expanded the endothelial cell population, suggesting that MGP is a factor in overall plasticity of adipose tissue.

**Conclusion:**

MGP displays a dual and cell-specific function in BAT, essentially creating a “cellular shuttle” that coordinates brown adipogenic differentiation with vascular growth during development.

## Highlights

• Matrix Gla protein (MGP) coordinates brown adipogenesis at two essential steps by regulating bone morphogenetic proteins.
• Endothelial-derived MGP limits triggering of white adipogenesis in brown adipose tissue.
• MGP derived from adipose cells supports transition of CD142+ cells to brown adipocytes.
• Reduction of MGP expands adipose capillary growth.

## 1 Introduction

Adipose tissue is commonly categorized into three types, white, beige, and brown adipose tissue, based on morphology, function and anatomical locations [1]. White adipose tissue (WAT) stores energy and supplies fuel. WAT is highly plastic, accounting for 3 %–70 % of overall body composition in well-trained lean athletes and morbidly obese individuals, respectively [2], and is mainly located in visceral and subcutaneous sites. In contrast, brown adipose tissue (BAT) is essential in thermoregulation [[3], [4], [5]], and helps maintain the body temperature through non-shivering thermogenesis [6,7].

BAT develops fully prior to birth but continues to grow, mainly due to cell proliferation until postnatal day (p)14, and thereafter by increasing the lipid content [8]. BAT is abundant in the interscapular and perirenal locations in human infants, whereas adult human BAT is found mainly in the cervical, supraclavicular and paravertebral regions [3,9]. In mice, the interscapular and perirenal BAT are composed of classical brown adipocytes, whereas beige adipocytes that are highly recruitable in “browning” of WAT are found in the subcutaneous anterior and inguinal depots [3,9]. BAT has been extensively discussed as a potential target for treatment of obesity and metabolic syndrome [3,10].

BAT is a highly vascularized tissue, which supports the function of thermogenesis [3]. Adipose tissue explants and adipocytes can trigger blood vessel formation, whereas endothelial cells (ECs) can promote preadipocyte differentiation [11,12]. In embryonic development, a stable capillary network precedes the differentiation of adipocytes [13]. In mouse gonadal fat, a dense vascular network expands quickly between p0 and p5, which gives rise to the adipocytes [14]. *In vitro*, human adipose endothelium promotes the proliferation of preadipocytes [15], and creates a vascular niche containing the progenitor cells [[16], [17], [18], [19]].

A hierarchy of adipose stem and progenitor cell (ASPC) populations has been proposed for the WAT that includes adipose stem cells (ASCs) (dipeptidyl peptidase-4 (DPP4)+, CD55+), pre-adipocytes (intercellular adhesion molecule 1 (ICAM1)+) and adipogenesis regulators (Aregs) (CD142+, also referred to as F3+) [1,20]. The expression pattern of *Dpp4* or platelet-derived growth factor receptor A (*Pdgfra*) in the ASCs suggests that ASCs are the most stem-cell like ASPCs, which are thought to give rise to ICAM1+ pre-adipocytes and CD142+ Aregs [20], potentially with interconversion between the two. It is not clear whether the hierarchy of ASPCs or individual cell types follow the same pattern in BAT.

The bone morphogenetic proteins (BMPs) play several important roles in adipogenesis [21,22] and angiogenesis [23]. White adipogenesis is enhanced by BMP2 and BMP4 [24], whereas BAT is dependent on BMP7 signaling for induction of appropriate brown adipogenesis [25,26], and drive human adipogenic stem cells into beige adipocytes [27]. BMP8b, closely related to BMP7, has also been shown to modulate thermogenesis in BAT and promote vascular sprouting [28,29]. *In vitro*, BMP4 and BMP7 are able to induce white to brown transition in murine and human white preadipocytes [30], whereas BMP7 promotes brown adipogenesis in brown preadipocytes and BMP4 suppresses expression of the thermogenic uncoupling protein 1 (*Ucp1*) [30].

Matrix Gla protein (MGP) is an efficient inhibitor of several BMPs including BMP2, BMP4 and BMP7 [[31], [32], [33]]. MGP is recognized as a vascular morphogen that is able to regulate both endothelial lineage, morphology, and the emergence of arterial calcification [[33], [34], [35], [36]]. Its function can be disrupted by warfarin interfering with the gamma-carboxylation of MGP [31], as well as genomic mutations and hereditary deficiencies [37]. Previous results indicated that MGP is expressed in human pre-adipocytes and associated with pre-adipogenic events in WAT, but decreases during adipocyte differentiation [38,39]. In addition, the expression is depot-dependent, with high expression in visceral adipose tissue [39,40]. Serum levels of inactive MGP has also been associated with visceral adipose tissue [39], suggesting that MGP may be involved in adipose regulation. However, its role in brown adipose development has not been reported.

In this study, we show that MGP has a dual and cell-specific function in BAT. Endothelial MGP limits whitening of BAT and supports brown adipogenesis, whereas progenitor cell-derived MGP promotes brown preadipocyte maturation.

## 2 Material and methods

*Key Resources* – see Supplemental Table 1.

### 2.1 Contacts for reagent and resource sharing

Requests for further information, resources, and reagents should be directed to corresponding authors Li Zhang (LiZ@mednet.ucla.edu) and Kristina I. Boström (kbostrom@mednet.ucla.edu). Sharing of primary samples is based on availability, and may be subject to Material Transfer Agreements (MTAs) and will require appropriate research ethics board certifications.

### 2.2 Animals

*Mgp*^*+/-*^ mice (B6.129S7-Mgptm1Kry/KbosJ, Stock No: 023811) Genotypes were confirmed by PCR [65], *Adipoq*^*Cre*^ (B6.FVB-Tg(Adipoq-cre)1Evdr/J, Stock No: 028020), *Adipoq*^*ERT2*^(C57BL/6-Tg(Adipoq-icre/ERT2)1Soff/J, Stock No: 025124), *mTmG* (B6.129(Cg)-*Gt(ROSA)26Sor*^*tm4(ACTB-tdTomato,-EGFP)Luo*^/J, Stock No: 007676), *VE-Cadherin*^*Cre*^ (B6.FVB-Tg(Cdh5-cre)7Mlia/J, Stock No: 006137). The *Mgp*^*flox/flox*^ (*Mgp*^*fl/fl*^*)* mouse was generated through our laboratory as previously described [66]. All mice were exposed to a standard 12h:12h light/dark cycles and fed a standard chow diet (Diet 8604; HarlanTeklad Laboratory). There were no significant differences in the phenotype or growth curves of female and male mice (Supplemental Figure 3). Therefore, male mice were included in this study. The studies were reviewed and approved by the Institutional Review Board and conducted in accordance with the animal care guidelines set by the University of California, Los Angeles. The investigation also conformed to the standards of National Research Council, *Guide of the Care and Use of Laboratory Animals, Eight Edition* (Washington, DC; The National Academies Press, 2011).

### 2.3 Body fat determination

Whole body fat, fluids, and lean muscle mass of mice were determined, each week one time, started from week one, using a Bruker Optics Minispec NMR analyzer (Billerica, MA) in accordance with the manufacturer's recommendations.

### 2.4 Stromal vascular fraction (SVF) cells

Isolation of SVF cells from interscapular brown adipose tissue (iBAT) was performed using a modification of methodology described previously [67]. Briefly, the iBAT was dissected, and the white adipose tissue (WAT) was carefully removed. The iBAT was subsequently minced and digested in 0.1 % (w/v) collagenase II solution (Sigma C6885) at 37 °C for 45 min with gentle agitation. Dulbecco's Modified Eagle Medium (DMEM) supplemented with 20 % fetal bovine serum (FBS) was used to stop the digestion. The digested iBAT was filtered through a 100 μm-cell strainer and centrifuged at 1000 rpm for 5 min; the pelleted layer contained the SVF and blood cells. Erythrocyte lysis buffer was used to disrupt red blood cells. The SVF cells were subsequently washed twice with FACS buffer (5 mm EDTA in 2 % BSA in Dulbecco's Phosphate-Buffered Saline (DPBS), pH 7.4).

### 2.5 Pre-BAT cells

The pre-BAT cells are a mouse brown pre-adipocyte cell line that was generously provided by the laboratory of Dr. Aldons J. Lusis at UCLA [68]. The pre-BAT cell line was derived from the SVF of mouse iBAT and immortalized by infecting the SVF with a retroviral vector expressing the SV40 T antigen. The pre-BAT cells were cultured as previously described [68] in growth medium containing 10 % FBS and 100 U/ml of both streptomycin and penicillin in high glucose DMEM. For adipocyte differentiation experiments, pre-BAT cells were plated in 12-wells plate at a density of 5 × 10^5^ cells per well. When the cells were almost 100 % confluent (day 0), the cells were treated with induction media, which consisted of growth media supplemented with 5 mg/mL insulin, 125 mM indomethacin, 1 nM 3,3’,5-Triiodo-l-thyronine (T3), 2 mg/mL dexamethasone, 0.5 mM 3-Isobutyl-1-methylxanthine (IBMX), and 0.5 mM Rosiglitazone for 48 h (day 0 to day 2). After 48 h, the cells were switched to maintenance medium, which consisted of the growth media supplemented with 5 mg/mL insulin, 1 nM T3, and 0.5 mM Rosiglitazone for 10 days (day 3 to day 12), with change of medium every 2 days.

### 2.6 ShRNA lentiviral particle transfection

MGP shRNA (sc-44627-v) and control shRNA (sc-108080), both from Santa Cruz Biotechnology, were transfected using 5 ug/ml Polybrene according to manufacturer's instructions. Puromycin was used for selection of stable clones. For determination of MGP shRNA knockdown efficiency, qRT-PCR was performed using primers listed in Table S1.

### 2.7 Fluorescence-activated cell sorting (FACS)

FACS analysis was performed as previously described [69]. See Supplemental Table 1 for antibody information. SVF cells from 4 weeks male iBAT were pooled and resuspended in FACS buffer (5 mm EDTA in 2 % BSA in DPBS, pH 7.4) for staining with following antibodies for 1 h at 4 °C in the dark: CD45-APC (1:100), CD31-PE (1:200), DPP4/CD26 Alexa Fluor 594 (1:100), ICAM1 Alexa Fluor 647 (1:100), and CD142 (1:100). The secondary antibodies used for CD142 were chicken anti-rabbit lgG (1:400) for 30 min. 4’,6-diamidino-2-phenylindole (DAPI, 1:5,000), which was used to stain dead cell, was added during the last 10 min. The SVF cells were washed three times with the FACS buffer to remove unbound antibodies. Flow cytometer gates were set using unstained cells as controls. Cells were gated by forward scatter versus side scatter to eliminate debris. DAPI-negative live cells were used for continued analysis. A minimum of 10,000 events were counted for each analysis. All FACS experiments were performed with a flow cytometer (LSR II; BD Biosciences). FACS results were exported and analyzed using FlowJoTMv10.7 software.

### 2.8 Vascular shunting

Fluorescent microspheres (15 μm FluoSpheres™, Invitrogen) were injected into the left ventricle immediately after sacrificing the mice, and iBAT was examined and photographed under fluorescence microscopy.

### 2.9 Immunoblotting

After euthanasia mice, the iBAT was immediately dissected and carefully separated from WAT. The tissues were frozen directly in liquid nitrogen and stored at −80 °C until use. Approximately 100 mg iBAT from three mice of the same genotype was homogenized in 800 μl RIPA buffer with phosphatase inhibitor (Roche, 1:100) and protease inhibitor (Roche, 1:100). Immunoblotting was performed as previously described [68] using nitrocellulose membranes. See Supplemental Table 1 for antibody information. The following primary antibodies from Cell Signaling Technology (CST) were diluted 1:1,000: FABP4, ACACA, FASN, Adiponectin, PPARγ, HSL, phospho(p)-HSL, SMAD1, phospho(p)-SMAD1/5, SMAD4. The primary antibodies against GAPDH from CST were diluted 1:5000. The primary antibodies against UCP1 from Abcam were diluted 1:2,000. The following day, the nitrocellulose membranes were washed with Tris-Buffered Saline with Tween (TBST) for 10 min each time, repeated 3 times, and incubated with horseradish peroxidase (HRP)-conjugated secondary antibodies for 1 h at room temperature. The nitrocellulose membranes were then washed 3 times in TBST, 10 min each time, and exposed in the ChemiDoc™ MP Gel Imaging System (Bio-Rad). The protein bands were quantified using ImageJ Software.

### 2.10 Immunofluorescence

Immediately after euthanasia using isoflurane, the mice were perfused with 50 ml of 1x phosphate-buffered saline (PBS) per mouse, and the iBAT was dissected and fixed in 4 % paraformaldehyde overnight. After washing twice with fresh water, the tissue was dehydrated and embedded in paraffin. Sections (5 μm) were cut from the paraffin blocks, and subsequently dewaxed, rehydrated and antigens were retrieved using unmasking solution. Sections were then incubated with primary antibodies. See Supplemental Table 1 for antibody information. The following antibodies were used: Perilipin1 (CST, 1:500), CD31 (R&D systems, 1:200), CD142 (Sino Biological, 1:50), MGP (Abcam, 1:2000), ICAM1 (Proteintech, 1:200), incubated at 4 °C overnight. Alexa Fluor–conjugated secondary antibodies (1:500, Invitrogen) were applied and the sections were co-stained with DAPI (Sigma–Aldrich, 1:5,000). Staining without antibodies and with lgG primary antibodies served as controls. Image acquisition was acquired with an inverted Nikon Eclipse Ti–S microscope (Nikon Corporation, Tokyo, Japan) or performed as Z-stacks using a LSM510 Meta confocal laser scanning microscope (Carl Zeiss, Jena, Germany) and the ZEN black software. Immunostaining was analyzed by ImageJ software.

### 2.11 Oil Red O and BODIPY™ 493/503 staining

After induction of pre-BAT cells to mature adipocytes, the cells were washed twice with 1xPBS, fixed in 4 % paraformaldehyde, and stained with Oil Red O as previously described [70]. To quantify the Oil Red O staining, the dye was eluted from the cells by 2-propanol, and the absorbance was measured at 518 nm. The 2-propanol served as the blank control. Staining with BODIPY™ 493/503 was performed after the same fixation as per the manufacturer's instructions.

### 2.12 BAT activity

Male mice of 4 weeks of age underwent cold treatment at 4 °C for 4 h as previously described [55]. Subsequently, each mouse received one injection of approximately 88 μCi of ^18^F-Fludeoxyglucose (^18^F-FDG) via the tail vein. An interval of 1 h for uptake was allowed between probe administration and micro-positron emission tomography (micro-PET)/micro-computed tomography (micro-CT) scanning. Image acquisition and reconstruction were performed as previously described [55]. Briefly, mice were placed in a dedicated imaging chamber designed for use for both the CT and PET systems. Data were acquired by using a GENXT PET/CT (Sofie Biosciences, Culver City, CA) for 15 min. PET and CT images were analyzed using Amide Software (version 1.16).

### 2.13 Electron microscopy

Fresh iBAT was cut to small pieces and fixed with 2.5 % glutaraldehyde and 3 % paraformaldehyde for 1 h at room temperature. The iBAT samples were prepared and imaged by transmission electron microscopy as previously described [71].

### 2.14 Quantitative reverse transcription polymerase chain reaction (RT-qPCR)

Total RNA was extracted from cells or iBAT using RNeasy mini kits (Qiagen) and RNeasy lipid tissue Mini Kits. CDNA was generated with high-capacity cDNA reverse transcription kits (Thermo Fisher Scientific) following the manufacturer's instructions. RT-qPCR was performed on a 7500 Fast Real-Time PCR System (Applied Biosystems) using TaqMan Universal PCR Master Mix (Thermo Fisher Scientific). Cycle conditions included one cycle at 50 °C for 2 min, one cycle at 95 °C for 10 min, and then 40 cycles at 95 °C for 15 s and 60 °C for 1 min. Threshold cycles of specific cDNAs were normalized to the housekeeping gene *Gapdh* and translated to relative values. See Supplemental Table 2 for primers used in qPCR.

### 2.15 RNA-seq library preparation and analysis

Total RNA from iBAT derived from 5 pairs of male global *Mgp* knock-out (*Mgp*-KO) and wild type mice was extracted using RNeasy micro-kits (Qiagen). RNA concentration and quality were assessed using NanoDrop™ 2000 (Thermo Fisher Scientific). Libraries were sequenced using the Illumina HiSeq 3000 System to a depth of approximately 20 million reads per library with single read of 50 base pairs. The reads were mapped with STAR 2.5.3a to the mouse genome (mm10) for the mouse cell libraries. The counts for each gene were obtained using --quantMode GeneCounts in STAR commands, and the other parameters during alignment were set to default. Counts normalized by sequencing depth were obtained using DESeq2 (version 1.38.3) estimateSizeFactors function with default parameters. Normalized counts were further log10 converted in R with command log10(x+1). Principle component analysis was performed on the log10 normalized counts using the R function prcomp with default parameters. Differential expression analyses were carried out on the raw counts using DESeq2 with default parameters. Genes with adjusted p value < 0.05 were considered as significantly differentially expressed. The differential expression genes (DEGs) were subjected to Gene Ontology (GO) enrichment analysis and molecular process analysis by g:Prolifer [45] and enrichR [46]. Heat maps were generated by Heatmapper [72].

### 2.16 Single cell RNA-seq analysis and pseudotime trajectory construction

Single cell RNA-seq data were acquired from Gene Expression Omnibus (GEO) database with accession number GSE207707. We used the datasets of stromal/stem cells (ASCs) and vascular cells derived from iBAT. The R package Seurat (v4.3.0) was used to cluster the cells and visualize the gene expression. Cells were first quality filtered to have more than 200 detected genes, less than 15,000 RNA counts and 10 % of mitochondrial genes. Data were transformed and regressed out the mitochondrial genes. The data from two replicates in room temperature condition were integrated and dimensional reduction, cell clustering, and data visualization were performed using t-stochastic neighboring embedding (tSNE). A subset of brown adipose progenitor cells and pre-adipocytes were further analyzed to construct single cell trajectories using the R package Monocle3 (v1.3.1). The cells were ordered along a learned trajectory and the expression dynamics of interested genes were plotted along the pseudotime.

### 2.17 Data availability

The RNA sequencing data sets of male iBAT from wild-type and *Mgp*-KO mice are accessible from GEO database with accession number GSE233274.

### 2.18 Statistics

Statistical analyses were performed using GraphPad InStat (version 9.0; Graph Pad Software Inc.). Data were analyzed by either unpaired two-tailed Student's *t* test or one way AVOVA with Tukey's multiple comparisons test for statistical significance. Data are represented as mean ± SEM. *P* values of less than 0.05 were considered significant, and experiments were repeated a minimum of 3 times.

## 3 Results

### 3.1 Mgp expression in BAT

**Figure 1:**
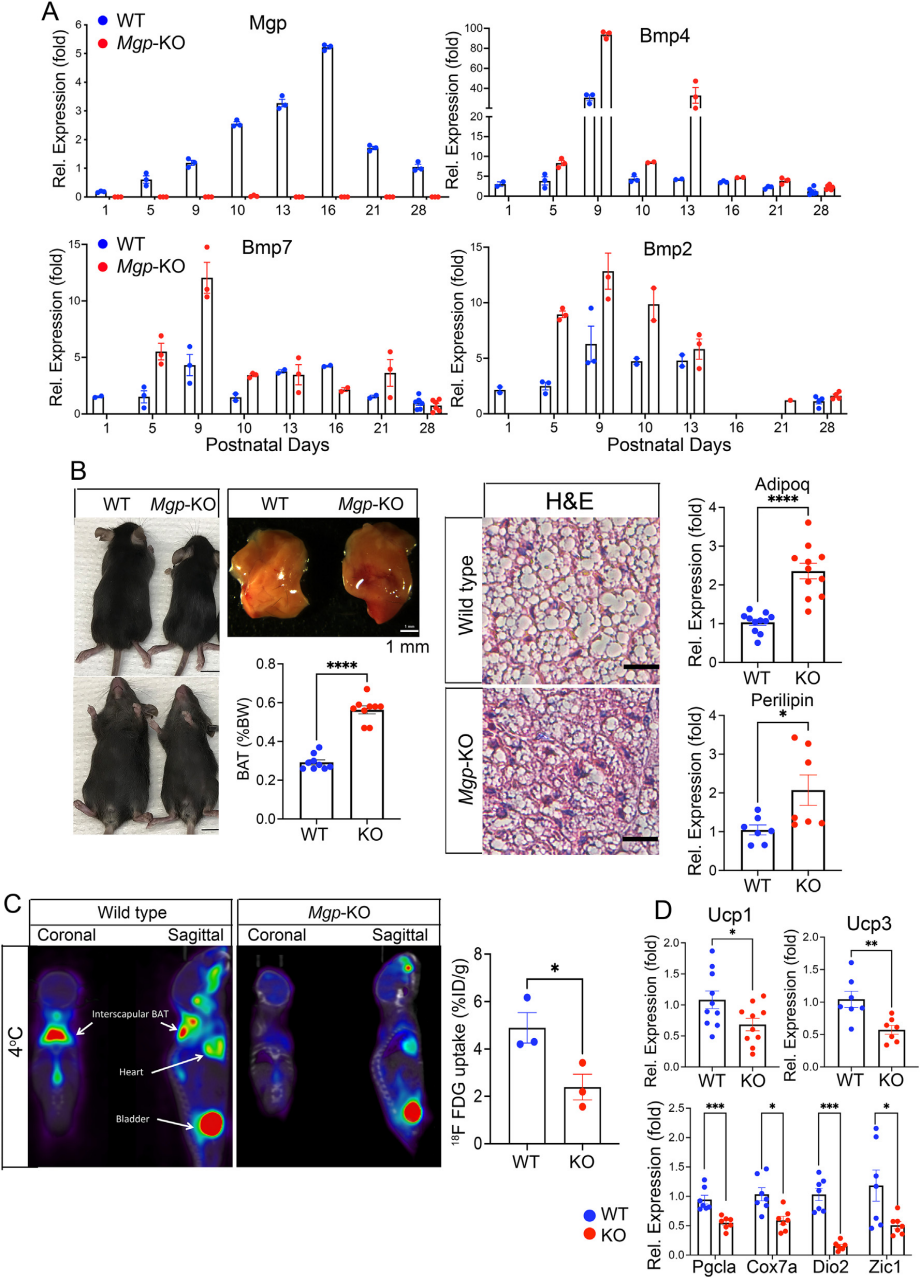
**Global MGP deficiency reduces brown adipose activity**. (**A**) Time course expression of *Mgp, Bmp4, Bmp7,* and *Bmp2* in iBAT from wild-type (WT) and global *Mgp*-knockout (KO) mice between postnatal day (p)1–28, as determined by qPCR. Expression is calculated as fold change compared to p1 (n = 3) **(B)** IBAT normalized to body weight in WT and KO mice at 4 weeks of age (left panels), H&E staining (middle panels; bars 25 μm) and expression of *Adipoq* and *Pln* in iBAT from WT and KO, as determined by qPCR (n ≥ 7 mice per group). (**C**) Reduced ^18^F-FDG uptake in KO mice as determined by ^18^F-FDG PET/CT and compared to wild-type (WT) mice (n = 3 mice per group). (**D**) Expression of the brown adipogenic markers *Ucp1*, *Ucp3*, *Pgcla*, *Cox7a*, *Dio7a* and *Zic1*, as determined by qPCR, in iBAT at 4 weeks of age (n ≥ 7 mice per group). Data are shown as mean ± SEM; (C, D, and E) unpaired two-tailed Student's *t* test, ∗p < 0.05, ∗∗p < 0.01, ∗∗∗p < 0.001, ∗∗∗∗p < 0.0001.

The BMPs are implicated in the development of BAT, with BMP7 being most strongly linked to brown adipogenesis [[25], [26], [27]]. However, it is relatively unknown whether individual BMP inhibitors such as MGP help coordinate the adipogenic process. To determine whether MGP plays a role in BAT, we first examined the MGP expression in the interscapular (i)BAT from wild-type and global *Mgp* knockout mice (*Mgp*-KO) mice between p1 to p28. Whereas no MGP was detected in the BAT of the *Mgp*-KO mice, expression of *Mgp* gradually increased and peaked around p16 in the wild-type mice (Figure 1A), which coincides with the end of the proliferative postnatal growth of BAT on p14 [8]. We also determined whether *Mgp* was expressed in the mature brown adipocytes using iBAT from *Adipo*^*CreERT*^*;mTmG* mice, where expression of the late adipocyte marker Adiponectin (*Adipoq*)-expressing cells abolish tdTomato expression but induce enhanced green fluorescent protein (eGFP) expression. The eGFP-positive mature brown adipocytes were isolated by FACS and confirmed to express *Mgp* by real-time PCR (qPCR) (Supplemental Figure 1 for experimental outline and results). We further examined expression of BMPs and BMP inhibitors reported in adipose tissue [21], including *Bmp2*, *Bmp4*, and *Bmp7*, which all peaked prior to *Mgp* (around day 9), and were enhanced by the *Mgp* deletion (Figure 1A). *Bmp6*, growth differentiation factor (*Gdf*)10 (also referred to as *Bmp3b*) and *Gdf5*, on the other hand, peaked at about the same time or later than *Mgp* and were less affected by *Mgp* deletion (Supplemental Figure 2A). *Bmp8a* and *Bmp8b* was not detected. The BMP inhibitors gremlin 1 (*Grem1*), Noggin (*Nog*), follistatin (*Fst*) and BMP and activity membrane bound inhibitor (*Bambi*) showed different patterns but were often enhanced in absence of MGP (Supplemental Figure 2A).

To determine if modulation of MGP influenced BMP-SMAD signaling during brown adipogenesis, we used the pre-BAT cells, an immortalized cell line derived from the iBAT stromal vascular fraction (SVF). The pre-BAT cells showed a gradual decrease of *Mgp* over 12 days while *Adipoq* was induced (Supplemental Figure 2B). We transfected the cells with *Mgp* shRNA, which reduced the MGP level to 10–15 % compared to control shRNA (Supplemental Figure 2C) and collected protein every two days for 12 days. Immunoblotting with densitometry showed a prolongation of the pSMAD1/5 activation when MGP was removed (Supplemental Figure 2D), supporting that MGP affects BMP signaling during early adipogenesis.

### 3.2 Global MGP deficiency alters the brown adipogenic phenotype

To examine the phenotype of the iBAT, we first focused on the global *Mgp*-KO mouse model. As previously reported, the *Mgp*-KO mice have limited life spans due to vascular disease [33,35]. When the *Mgp*-KO mice were allowed an *ad libitum* chow diet for up to 4 weeks, the percent fat gradually decreased, the percent lean mass increased, and the body weight remained about the same in both females and males (Supplemental Figure 3). Despite the gradual decrease in percent fat, we discovered that the *Mgp*-KO mice had an approximately 2-fold greater proportion of iBAT normalized to body weight.

The iBAT from the Mgp-KO mice showed enhanced expression of the adipocyte markers *Adipoq* and Perilipin (*Pln*), but less lipid accumulation as assessed by H&E staining (Figure 1C). Furthermore, the thermogenic activity was reduced in the *Mgp*-KO mice compared to wild-type controls at 4 weeks of age, as determined by ^18^F-FDG uptake at 4 °C (Figure 1D). A reduction was also observed in the expression of thermogenesis-related genes, including *Ucp1*, *Ucp3*, peroxisome proliferator-activated receptor-γ coactivator-1α (*Pgc1a*), cytochrome c oxidase subunit 7A (*Cox7a*), iodothyronine deiodinase 2 (*Dio2*) and zic family member 1 (*Zic1*), in the *Mgp*-KO mice (Figure 1E). Furthermore, electron microscopy revealed an increased number of similar-sized mitochondria in the *Mgp*-KO iBAT (Supplemental Figure 4A,B) but with minor changes in the expression of the mitochondrial transcription factor A (*Tfam*) and the nuclear respiratory factor 1 (*Nrf1*) (Supplemental Figure 4C). The results were consistent with a disruption of the normal brown adipogenic differentiation. Interestingly, the *Mgp* deletion also disrupted the aortic perivascular adipose tissue (PVAT), which mainly comprises BAT. It showed ^18^F-FDG uptake in the wild-type mice but not in the *Mgp*-KO mice (Figure 1D), and was more vascularized with less adipose elements (Supplemental Figure 5).

### 3.3 Global Mgp deficiency disrupts brown adipogenic maturation

**Figure 2:**
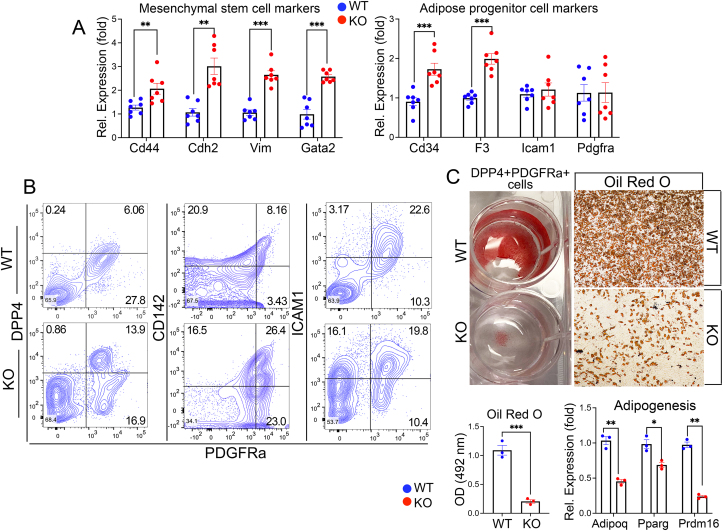
**Global MGP deficiency disrupts brown adipogenic maturation**. (**A**) Expression of the mesenchymal stem cell markers *Cd44*, *Cdh2*, *Vim* and *Gata2* (left panel) and the adipose progenitor cell markers *Cd34*, *Cd142*, *Icam1* and *Pdgfra* (right panel), in wild-type (WT) and *Mgp*-knockout (KO) iBAT, as determined by qPCR (n = 7 mice per group). (**B**) FACS analysis of adipose progenitor cells from the brown adipose stromal vascular fractions isolated from WT and KO mice (n = 5 mice per group), using antibodies to ICAM1, CD142, DPP4 and PDGFRA. (**C**) PDGFRA + DPP4+ double-positive cells from the stromal vascular fractions were cultured in adipogenic conditions for 12 days. The cells were stained with Oil Red O or collected for RNA analysis. The Oil Red O was extracted and quantified at 492 nM. Expression of *Adipoq*, *Pparg* and *Prdm16* was determined by qPCR (n = 3; representative of 3 replicate experiments). Data are shown as mean ± SEM; (A and C) unpaired two-tailed Student's *t* test, ∗p < 0.05, ∗∗p < 0.01, ∗∗∗p < 0.001, ∗∗∗∗p < 0.0001.

MGP inhibits BMP4 [31], which is known to promote proliferation and commitment of early mesenchymal stem cells to preadipocytes [41]. To determine if the loss of MGP influenced the brown progenitor stage, we compared the lineage markers in *Mgp*-KO and wild-type iBAT. We found that the expression of both the mesenchymal stem cell markers *Cd44*, cadherin 2 (*Cdh2*), vimentin (*Vim*), GATA-binding factor 2 (*Gata2*) and the adipose progenitor cell markers *Cd34*, *Cd142*, *Icam1* and *Pdgfra* was increased in the *Mgp*-KO iBAT compared to wild type (Figure 2A). Whereas *Pdgfra* is widely expressed in progenitor cells, *Dpp4* is considered an early adipose progenitor marker, preceding the expression of *Cd142* and *Icam1* [1,20]. To determine the effect of MGP-deficiency on the different cell types, we isolated PDGFRA + cells that were double-positive for DPP4, CD142 or ICAM1 from *Mgp*-KO and wild-type iBAT. The FACS analysis showed that the *Mgp*-KO iBAT had 13.9 % PDGFRA + DPP4+ cells versus 6.06 % in wild type (Figure 2B). The fraction of PDGFRA + CD142+ cells was enhanced at 26.4 % PDGFRA + CD142+ cells versus 8.16 % in wild type, whereas only a minor change was seen in the PDGFRA + ICAM1+ cells. Together, it points to a delay or re-direction of primarily PDGFRA + CD142+ and PDGFRA + DPP4+ cells. To assess the adipogenic capacity of the DPP4+ cells *in vitro*, we isolated PDGFRA + DPP4+ cells by FACS from *Mgp*-KO and wild-type iBAT, and seeded an equal number of cells in adipogenic conditions for 12 days. Oil Red O staining showed that the MGP-deficient PDGFRA + DPP4+ cells had less lipid accumulation as well as reduced expression of the adipogenic genes *Adipoq*, peroxisome proliferator-activated receptor γ (*Pparg*), and PR domain containing 16 (*Prdm16*) (Figure 2C) compared to wild-type PDGFRA + DPP4+ cells, further supporting the disruption of brown adipogenesis in MGP-deficiency. Despite the reduction in lipid accumulation, there was no reduction in cell numbers, as evidenced by combined BODIPY and DAPI stains (Supplemental Figure 6).

### 3.4 Global Mgp deficiency dysregulates adipose vascular formation

**Figure 3:**
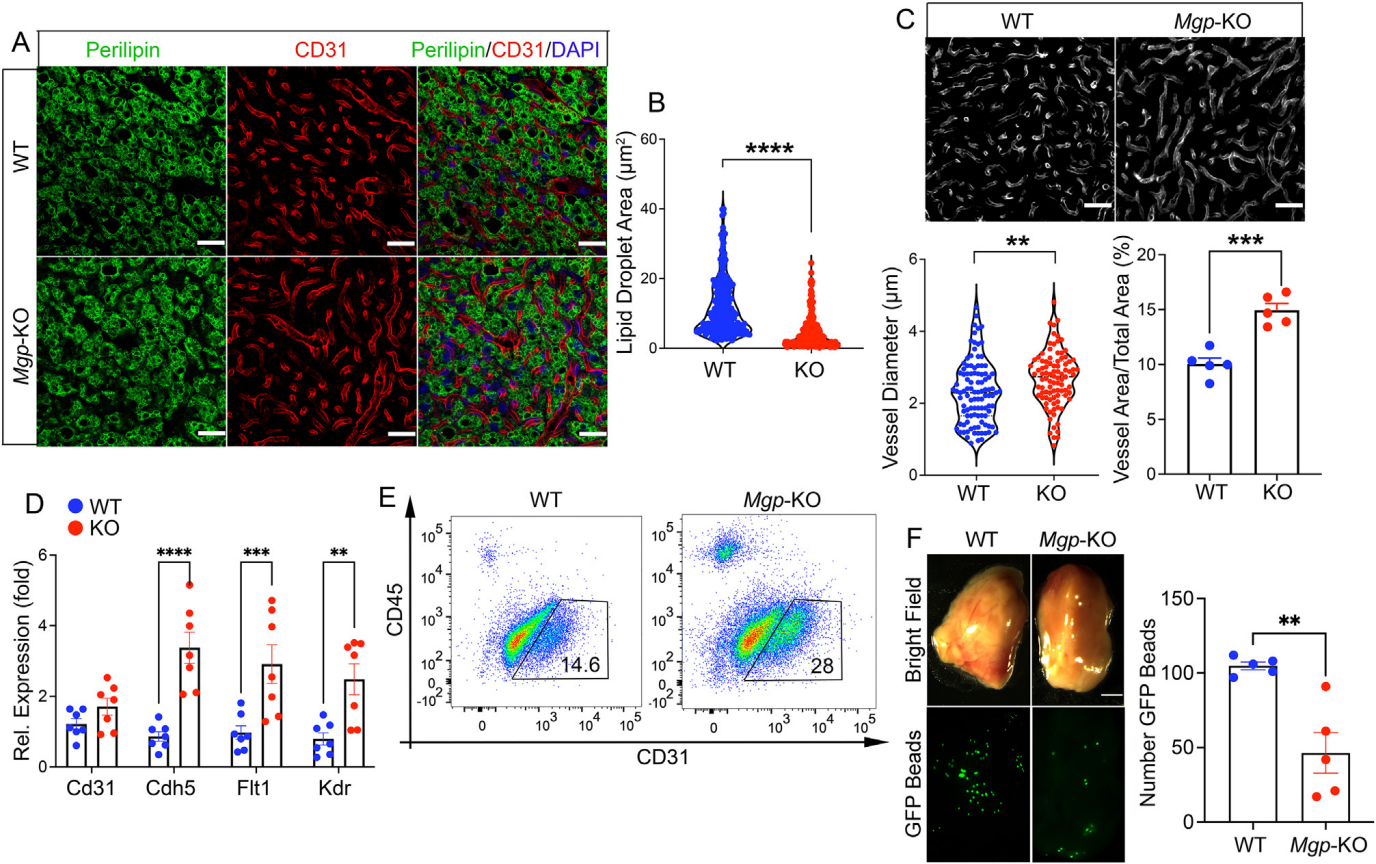
**Global MGP deficiency disrupts vascular formation in iBAT**. (**A**) Immunofluorescence for Perilipin (green) and CD31 (red) in iBAT from wild-type (WT) and *Mgp*-knockout (KO). DAPI (blue) was used to visualize nuclei. Bars, 12.5 μm. (**B**) Reduced lipid droplet area in KO iBAT (n = 5 mice per group). (**C**) Increased vessel diameter and total vessel area in KO iBAT as determined by ImageJ after staining of iBAT for CD31 and conversion of images to black and white. (**D**) Expression of the endothelial markers *Cd31*, *Cdh5*, *Flk1* and *Kdr* in WT and KO iBAT as determined by qPCR (n = 7 mice per group). (**E**) FACS analysis of CD31+ ECs from the brown adipose stromal vascular fractions isolated from WT and KO mice (n = 5 per group). (**F**) Evidence of AV shunting in iBAT, as assessed after injection of green fluorescent protein (GFP) microspheres (15 μm) followed by visualization under bright field and green fluorescent light (bars, 1 mm). The microspheres were counted and plotted (n = 5 retinas per group). Data are shown as mean ± SEM. unpaired two-tailed Student's *t* test, ∗∗p < 0.01, ∗∗∗p < 0.001, ∗∗∗∗p < 0.0001.

Since MGP is an effective regulator of angiogenesis and endothelial cell (EC) differentiation [34,42], we examined the capillary formation in iBAT from *Mgp*-KO mice and wild type. The percent CD31^+^ vessel area and average capillary diameter were 2.7 μm and 14.9 %, respectively, in the *Mgp*-KO iBAT, compared to 2.3 μm and 10.0 % in wild type, as estimated by immunofluorescence (Figure 3A,C). Perilipin staining suggested a corresponding reduction in lipid droplet size to 4.4 μm^2^ in the *Mgp*-KO iBAT, compared to 11.7 μm^2^ in wild type (Figure 3A,B). In addition, the expression of the endothelial markers fms-like tyrosine kinase 1 (*Flt1*), kinase insert domain receptor (*Kdr*), and VE-cadherin (*Cdh5*) increased after loss of MGP (Figure 3D). FACS analysis of CD31+CD45- ECs showed that the *Mgp*-KO iBAT contained 28 % CD31+CD45- ECs compared to 14.6 % in wild-type iBAT (Figure 3E). Since loss of MGP is known to cause arteriovenous malformations in organs such as the brain, lungs, kidneys, and retina [33,43,44], we tested if arteriovenous (AV) shunting was also present in iBAT. We performed functional assays by injecting fluorescent microspheres and determining the number of beads retained in the iBAT [44]. The results showed that the number of retained beads was reduced to about 30 % of normal in the *Mgp*-KO iBAT (Figure 3F) indicating abnormal and enlarged capillaries in the MGP-deficient iBAT.

### 3.5 MGP-deficiency promotes stem cell characteristics in iBAT as per RNA-Seq analysis

To compare the transcriptional profiles of MGP-deficient versus normal iBAT, we performed genome-wide RNA-Seq of iBAT from 5 pairs of male *Mgp*-KO and wild-type mice. In the 27,268 mapped genes, we identified 1,138 differentially expressed genes (DEGs; FDR <0.05) in the *Mgp*-KO mice, including 604 down-regulated and 534 up-regulated genes (Supplemental Figure 7A). Using the g:Profiler tool [45], enrich R and GSEA [46], we identified significantly enriched gene ontology (GO) terms for these DEGs. The result revealed that metabolic process, brown fat cell differentiation and triglyceride metabolic process decreased after *Mgp* deletion, whereas cell proliferation and migration, developmental process and blood vessel development increased (Supplemental Figure 7B and 8A), consistent with a promotion of stem cell-associated characteristics and delayed maturity.

The RNA-seq showed that expression of genes related to adipogenesis and metabolism, including adiponectin receptor 2 (*Adipor2*), perilipin 2 (*Pln2*), fatty acids synthase (*Fasn*), acyl-CoA synthetase long chain family member 5 (*Acsl5*), fatty acid elongase 5 (*Elovl5*), stearoyl-CoA 9-desaturase (*Scd1*), and acetyl-CoA carboxylase α (*Acaca*), declined in the *Mgp*-KO iBAT as illustrated by the heat map (Supplemental Figure 7C) and volcano plot with confirmation by qPCR (Supplemental Figure 8B). On the other hand, gene expression related to cell migration and developmental process, including *Bmp4*, Delta-like canonical Notch ligand 4 (*Dll4)*, Cbp/P300 interacting transactivator 2 (*Cited2*), Copine family member 9 (*Cpne9*), BOI-related gene 2 (*Btg2*), proto-oncogene *Fos*, and zinc finger protein 36 (*Zfp36*), increased in the *Mgp*-KO iBAT. RNA and protein levels were confirmed for selected genes by qPCR and immunoblotting. *Fasn*, *Scd1* and *Acaca*, related to lipid biosynthesis, were reduced in the *Mgp*-KO iBAT (Supplemental Figure 7D,E). However, *Fos*, Ras association domain family member 4 (*Rassf4*), early growth response protein 1 (*Egr1*) *Btg2*, *Capn9*, skeletal α-actin (*Acta1*), and vasorin (*Vasn*), related to cell migration and development, all increased (Supplemental Figure 8C). Thus, MGP is essential for normal adipogenic maturation.

### 3.6 Mgp is highly expressed in part of the ECs and adipose progenitor cells

**Figure 4:**
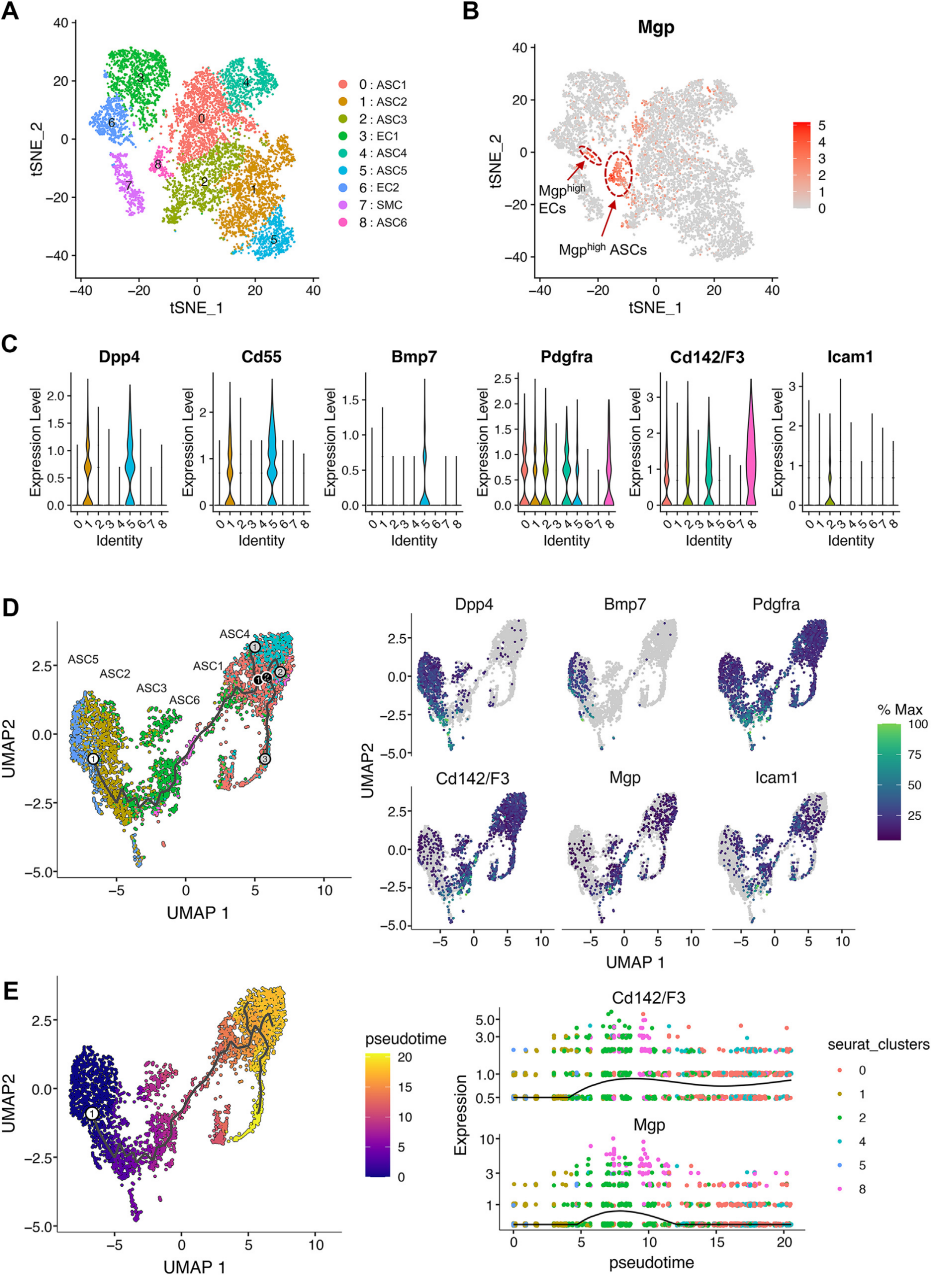
**Single cell RNA-seq data analysis of GEO dataset GSE207707**. (**A**) t-Stochastic neighboring embedding (tSNE) plot of brown adipose stromal/stem cells (ASCs) and vascular cells. EC, endothelial cells; SMC, smooth muscle cells. (**B**) Scatter plot of *Mgp* expression in brown adipose ASCs and vascular cells. (**C**) Violin plots of the marker genes of brown adipose progenitor cells and pre-adipocytes. (**D**) Single-cell trajectory of brown adipose ASCs and the expression of marker genes along the trajectory. (**E**) Brown adipose ASCs ordered by pseudotime along the single-cell trajectory and gene expression dynamics of *Mgp* and *Cd142/F3* in the ASC differentiation course.

We then examined the expression pattern of *Mgp* in adipose ECs, adipose progenitor cells, and preadipocytes by analyzing the publicly available single-cell (sc)RNA-seq datasets from mouse BAT (Gene Expression Omnibus (GEO) dataset GSE207707) [47,48]. In the scRNA-seq analysis, ASCs from mouse iBAT were clustered similarly to what has been shown in WAT, with two populations of *Dpp4*, *Cd55*, and *Bmp7* expressing adipose stem and progenitor cells, ASC2 and ASC5; and four populations of *Cd142* and *Icam1* expressing pre-adipocytes, ASC1, ASC3, ASC4 and ASC6 (Figure 4A). Expression of *Pdgfra* was observed in all ASCs. The data showed that *Mgp* was highly expressed in a sub-population of ECs (EC6) and in CD142+ Aregs (ASC6) (Figure 4B). In addition, *Mgp* was moderately expressed in ICAM1+ pre-adipocytes (ASC3) (Figure 4B). We verified that MGP co-localized with CD31 (ECs) and ICAM1 (ASCs) in iBAT from 4-week old mice by immunofluorescence (Supplemental Figure 9). In all, *Mgp* expression was limited to sub-populations of ECs and adipose cells, rather than a general *Mgp* expression in all cell types.

To elucidate the role of *Mgp* in adipogenesis progression, we performed pseudotime trajectory analysis using R Package Monocle 3. A similar trajectory to WAT from young mice were observed in BAT. Most interestingly, brown adipose ASCs ordered by pseudotime along the trajectory and gene expression dynamics showed that *Mgp*^*high*^ *Cd142/F3*^*high*^ cells (ASC6) were bridging the differentiation from ASPCs to pre-adipocytes (Figure 4D,E), suggesting a role for MGP in the transition of CD142+ cells to maturity.

### 3.7 Cell-specific Mgp deletion in endothelial and adipose cells

**Figure 5:**
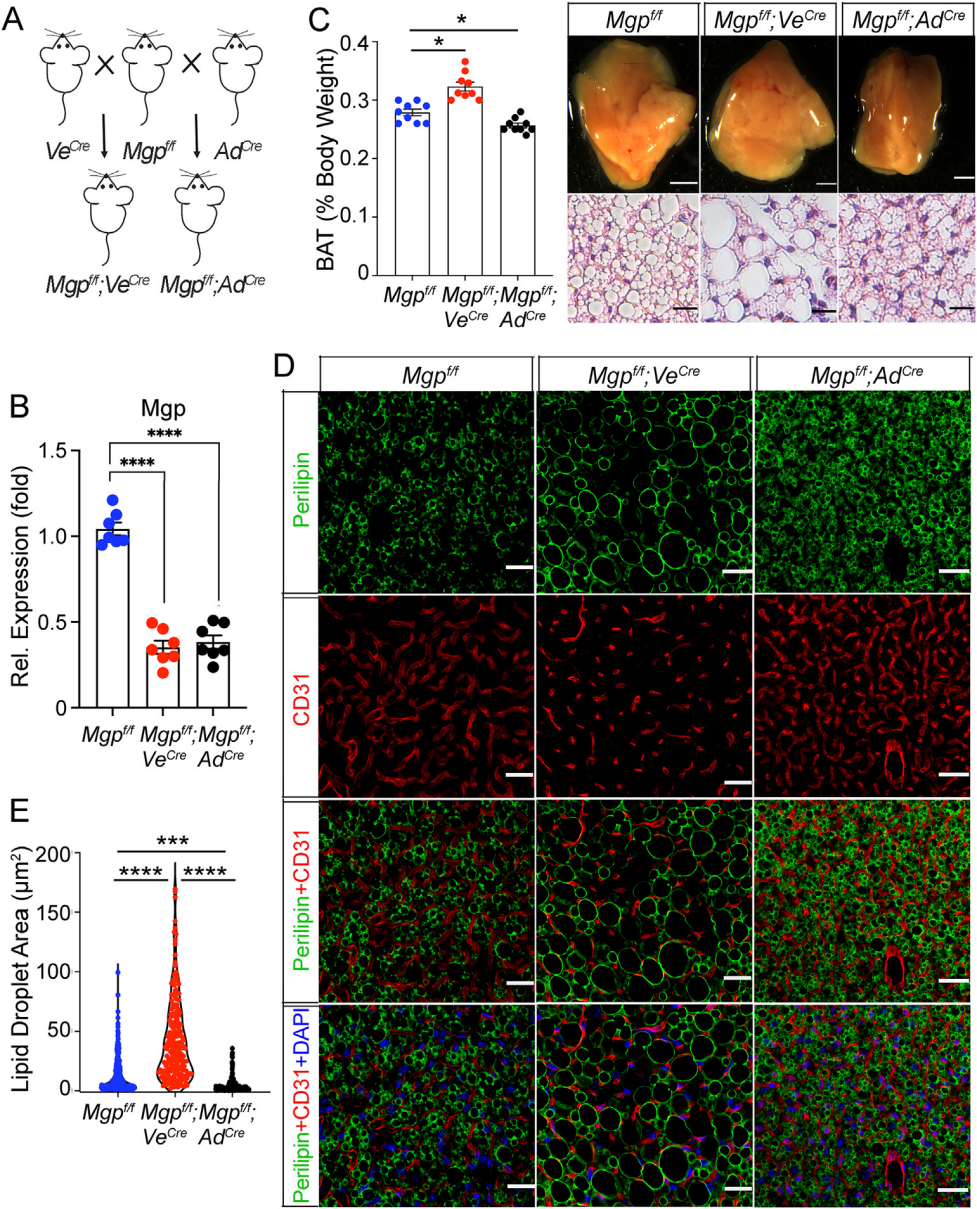
**Cell-specific Mgp deletions in endothelial and adipose cells promote whitening versus disruption of brown adipogenic differentiation.** (**A**) Schematic diagram of breeding strategy. (**B**) Expression of *Mgp* in iBAT, as determined by qPCR (n = 7 mice per group). (**C**) (Left panel) Percent BAT of body weight in the three mice (n = 9 mice per group).) (Right panel) Bright field and H&E staining of iBAT derived from control *Mgp*^*f/f*^ mice, *Mgp*^*f/f*^*; Ve*^*Cre*^ mice, and *Mgp*^*f/f*^*; Ad*^*Cre*^ mice. Bars, 1 mm (top), 12.5 μm (bottom). (**D**) Immunofluorescence for Perilipin (green) and CD31 (red) in iBAT from *Mgp*^*f/f*^ mice, *Mgp*^*f/f*^*; Ve*^*Cre*^ mice, and *Mgp*^*f/f*^*; Ad*^*Cre*^ mice. DAPI was used to visualize the nuclei. Bars, 12.5 μm (representative of 3 replicate experiments). (**E**) Lipid droplet area in iBAT from *Mgp*^*f/f*^ mice, and *Mgp*^*f/f*^*; Ve*^*Cre*^ and *Mgp*^*f/f*^*; Ad*^*Cre*^ mice as determined by ImageJ after staining of iBAT for Perilipin (data from n = 3 mice per group). Data are shown as mean ± SEM; One way ANOVA, ∗p < 0.05, ∗∗p < 0.01, ∗∗∗p < 0.001, ∗∗∗∗p < 0.0001.

To identify the potential role of MGP in both endothelium and ASCs, we deleted *Mgp* in the endothelium using *Mgp*^*f/f*^ mice crossbred with the *VE-Cadherin Cre (Ve*^*cre*^*)* transgenic mice (Figure 5A for breeding strategy), thereby reducing the amount of MGP in the vicinity of the adipose progenitor cells in the perivascular area. To abolish MGP in the brown adipocytes, previously confirmed to express *Adipoq* (Supplemental Figure 1), we deleted *Mgp* using the *Adipoq Cre (Ad*^*cre*^*)* transgenic mouse. Each deletion caused a reduction in Mgp expression in iBAT to about a third of the *Mgp*^*f/f*^ controls (Figure 5B). There were no significant changes in body weight or percent lean mass of the cell-specific deletions. A minor reduction in percent fat was observed in the *Mgp*^*f/f*^*; Ad*^*cre*^ mice (Supplemental Figure 10A).

In *Mgp*^*f/f*^*;Ve*^*cre*^ mice, the percent iBAT of total body weight increased significantly compared to the *Mgp*^*f/f*^ control*s* (Figure 5C). Histologically, the size of the lipid droplets increased by H&E staining and Perilipin immunofluorescence (Figure 5C,D), and showed a significant increase in the lipid droplet area (Figure 5E) compared to *Mgp*^*f/f*^ controls. This is consistent with a “whitening” of the BAT, suggesting that MGP limits diversion to white adipogenic differentiation in the perivascular area. In the *Mgp*^*f/f*^*; Ad*^*cre*^ mice, however, the percent iBAT of total body weight decreased compared to controls (Figure 5C). The size of the brown adipocytes was reduced with less lipid accumulation as assessed by H&E staining and Perilipin immunofluorescence (Figure 5C,D). The total lipid droplet area was reduced compared to *Mgp*^*f/f*^*;Ve*^*cre*^ and *Mgp*^*f/f*^ iBAT (Figure 5E), suggesting a separate role for MGP in balancing lipolytic activity. Interestingly, similar changes in the size of the lipid droplets were seen in response to *Mgp* deletion in the inguinal WAT, and to a lesser degree in the gonadal WAT (Supplemental Figure 10B,C).

### 3.8 Cell-specific loss of MGP affects differentiation and thermogenic function

**Figure 6:**
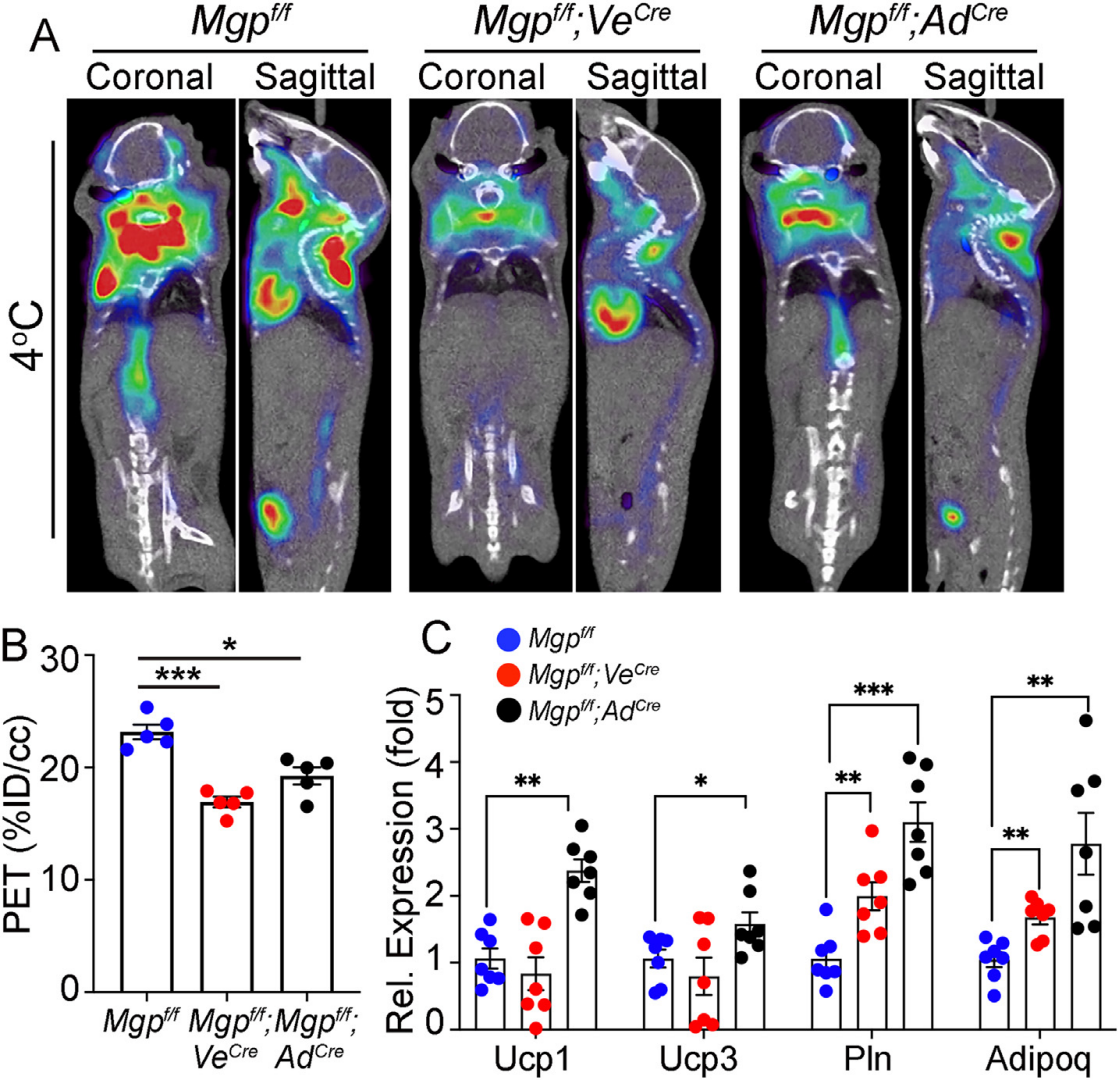
**Cell-specific Mgp deletions reduce brown adipogenic activity.** (A, B) Reduced ^18^F-FDG uptake in *Mgp*^*f/f*^*; Ve*^*Cre*^ and *Mgp*^*f/f*^*; Ad*^*Cre*^ mice, as determined by ^18^F-FDG PET/CT and compared to *Mgp*^*f/f*^ mice (n = 5 mice per group, representative image shown). (C) Expression of *Ucp1*, *Ucp3*, *Pln* and *Adipoq* in *Mgp*^*f/f*^ mice, *Mgp*^*f/f*^*; Ve*^*Cre*^ and *Mgp*^*f/f*^*; Ad*^*Cre*^ mice, as determined by qPCR (n = 7 mice per group). Data are shown as mean ± SEM; One way ANOVA, ∗p < 0.05, ∗∗p < 0.01, ∗∗∗p < 0.001.

We compared lineage marker expression in the different iBATs and found that the expression of the common adipocyte markers *Pln1* and *Adipoq* was increased in both *Mgp*^*f/f*^*;Ve*^*cre*^ and *Mgp*^*f/f*^*;Ad*^*cre*^ mice compared to *Mgp*^*f/f*^ controls (Figure 6A). However, the expression of the brown-specific thermogenic genes *Ucp1* and *Ucp3* was increased in the *Mgp*^*f/f*^*; Ad*^*cre*^ iBAT but unaffected in the *Mgp*^*f/f*^*;Ve*^*cre*^ iBAT compared to controls (Figure 6A). Functionally, both *Mgp*-deletions caused a suppression in brown adipose activity as determined by ^18^F-FDG PET/CT (Figure 6B,C), further supporting that MGP is required in both cell types for optimal BAT function.

**Figure 7:**
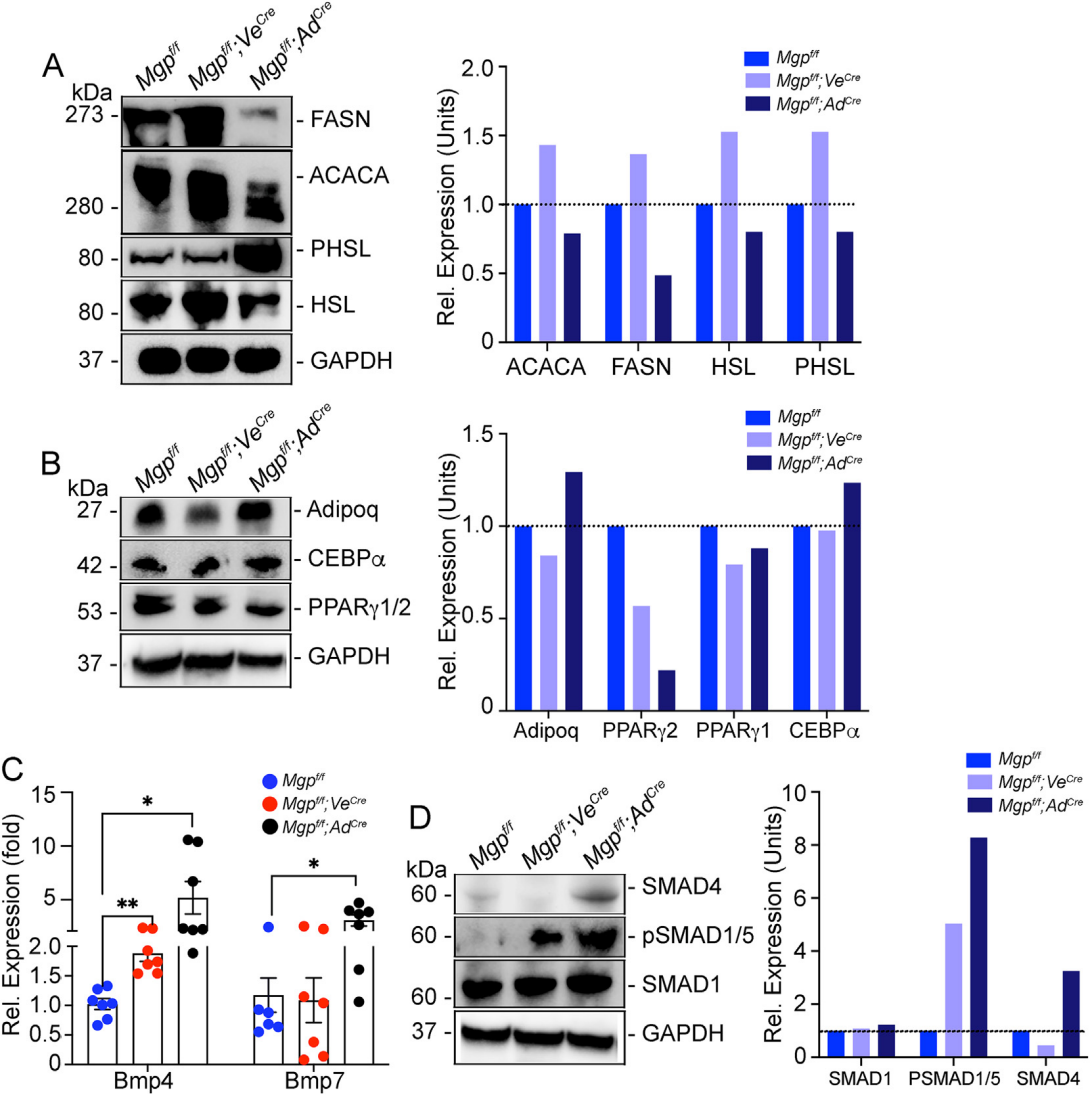
**Marker expression and SMAD activation after endothelial and adipose Mgp deletion.** (**A**) Expression of FASN, ACACA, HSL and P-HSL in iBAT from *Mgp*^*f/f*^, *Mgp*^*f/f*^*; Ve*^*Cre*^, and *Mgp*^*f/f*^*; Ad*^*Cre*^ mice, as determined by immunoblotting and quantified by densitometry. (**B**) Expression of Adiponectin, PPARg1/2 and CEBPa in iBAT, as determined by immunoblotting and quantified by densitometry. (**C**) Expression of *Bmp4* and *Bmp7* in iBAT, as determined by qPCR (n = 7 mice per group). (**D**) Expression of SMAD4, pSMAD1/5, SMAD1 in iBAT, as determined by immunoblotting and quantified by densitometry. The immunoblots were representative of 3 replicate experiments; GAPDH was used as loading control. Data from qPCR are shown as mean ± SEM; One way ANOVA, ∗p < 0.05, ∗∗p < 0.01, ∗∗∗p < 0.001, ∗∗∗∗p < 0.0001.

The whitening of the *Mgp*^*f/f*^*;Ve*^*cre*^ BAT was associated with enhanced expression of *Fasn* and *Acaca* (Figure 7A), as determined by immunoblotting, which supported enhanced lipid biosynthesis. The activation (phosphorylation) of hormone sensitive lipase (pHSL), which is rate-limiting step in lipolysis, was similar to the *Mgp*^*f/f*^ control (Figure 7A). However, pHSL was highly activated in the *Mgp*^*f/f*^*Ad*^*cre*^ mice, while FASN and ACACA were suppressed (Figure 7A). The level of the adipogenic markers Adipoq, CCAAT/enhancer-binding protein α (CEBPα), and PPARγ1 remained about the same, whereas PPARγ2 decreased with the *Mgp* deletion (Figure 7B). Together, it is consistent with the enhanced versus diminished lipid accumulation in the *Mgp*^*f/f*^*;Ve*^*cre*^ and *Mgp*^*f/f*^*; Ad*^*cre*^ iBAT, respectively.

### 3.9 Marker expression and SMAD activation after endothelial and adipose Mgp deletion

We then examined if the cell-specific *Mgp* deletions had affected the expression of *Bmp4* and *Bmp7*, and BMP signaling (Figure 7C,D). The results showed that Bmp4 alone increased in the *Mgp*^*f/f*^*;Ve*^*cre*^ iBAT as determined by qPCR, whereas both Bmp4 and Bmp7 increased in the *Mgp*^*f/f*^*;Ad*^*cre*^ iBAT (Figure 7C). Immunoblotting showed an increase in activated, phosphorylated (p)SMAD1/5 in both mice compared to SMAD (Figure 7D) consistent with enhanced BMP signaling. Furthermore, the level of SMAD4 was enhanced in the *Mgp*^*f/f*^*;Ad*^*cre*^ mice, further promoting BMP signaling.

### 3.10 Cell-specific loss of MGP promotes endothelial expansion

**Figure 8:**
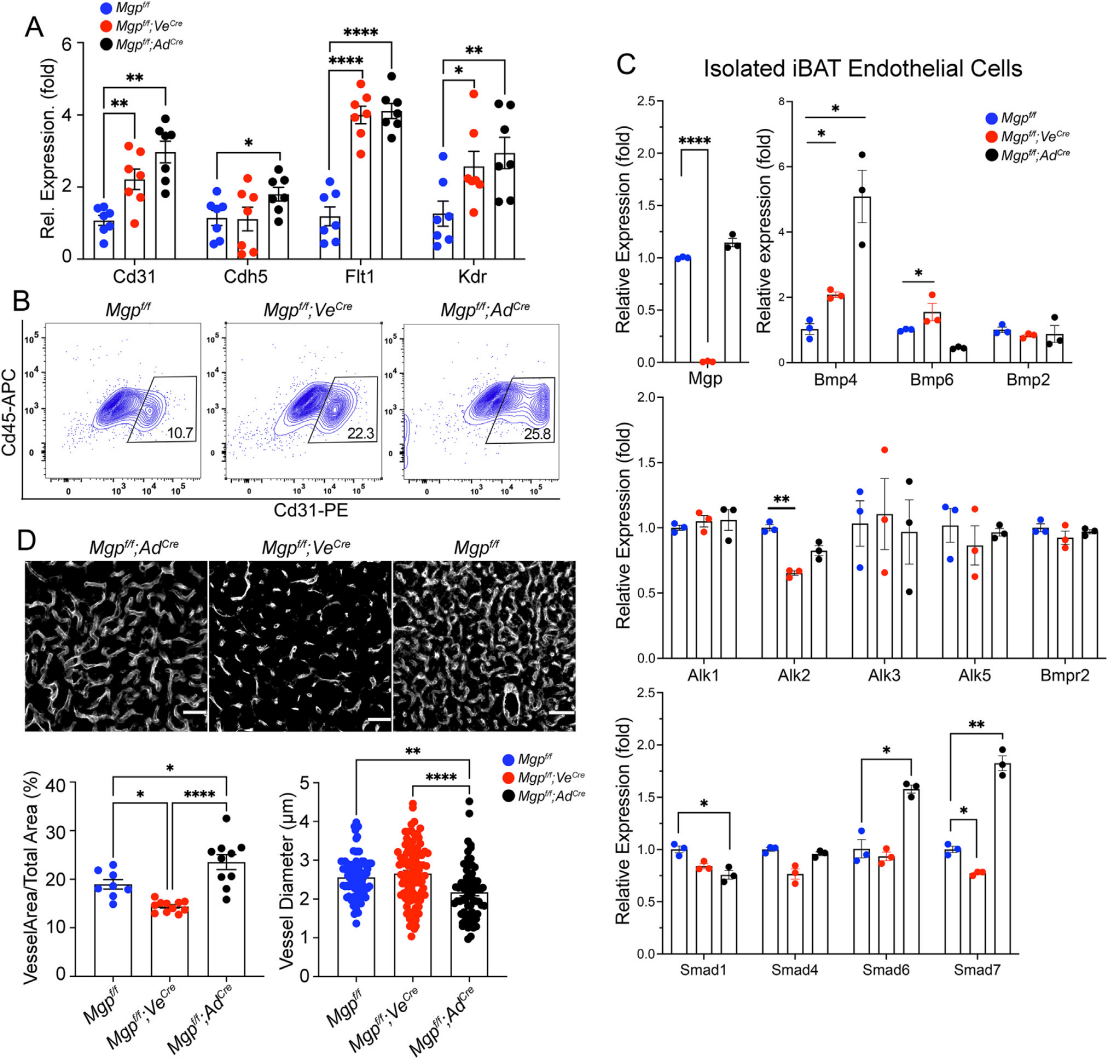
**Both endothelial and adipose Mgp deletion expands vascular growth.** (**A**) Expression of *Cd31*, *Cdh5*, *Flt1* and *Kdr* in iBAT from *Mgp*^*f/f*^*, Mgp*^*f/f*^*; Ve*^*Cre*^*and Mgp*^*f/f*^*; Ad*^*Cre*^ mice, as determined by qPCR (n = 7 mice per group). (**B**) FACS analysis of CD45-and CD31+ endothelial cells from the brown adipose stromal vascular fractions isolated from the three groups of mice (n = 5 mice per group). (**C**) Expression of *Mgp, Bmp4, Bmp6, Bmp2, Alk1, Alk2, Alk3, Alk5, Bmpr2, Smad1, Smad4, Smad 6,* and *Smad7* in endothelial cells isolated from iBAT in *Mgp*^*f/f*^ mice, *Mgp*^*f/f*^*: Ve*^*Cre*^ mice, and *Mgp*^*f/f*^*: Ad*^*Cre*^ mice (4 weeks of age, data from combined n = 3–4 mice), as determined by qPCR. Expression is calculated as fold change compared to the expression in *Mgp*^*f/f*^ mice. (**D**) Vascular pattern as assessed after immunostaining for CD31 (red converted to white). Bars, 12.5 μm. Vessel area and vessel diameter in iBAT from *Mgp*^*f/f*^, *Mgp*^*f/f*^*; Ve*^*Cre*^ and *Mgp*^*f/f*^*; Ad*^*Cre*^ mice as determined by ImageJ after conversion of images to black and white. Data are shown as mean ± SEM; One way ANOVA, ∗p < 0.05, ∗∗p < 0.01, ∗∗∗∗p < 0.0001.

The cell-specific *Mgp* gene deletions enhanced the adipose vasculature. The endothelial markers *Cd31*, *Cdh5*, *Flt1*, and *Kdr* increased in both *Mgp*^*f/f*^*;Ve*^*cre*^ and *Mgp*^*f/f*^*;Ad*^*cre*^ iBAT (Figure 8A), and FACS revealed an increase in CD31+CD45- ECs from 10.7 % in *Mgp*^*f/f*^ control mice, to 22.3 % and 25.8 % in *Mgp*^*f/f*^*;Ve*^*cre*^ and *Mgp*^*f/f*^*;Ad*^*cre*^ iBAT, respectively (Figure 8B), pointing to enhanced endothelial expansion. We then isolated ECs from each iBAT. We confirmed the lack of *Mgp* expression in the ECs from the *Mgp*^*f/f*^*;Ve*^*cre*^ iBAT (Figure 8C). The *Mgp* deletion in either the ECs or the adipose cells enhanced the expression of BMP4 in the ECs (Figure 8C). Since BMP4 stimulates EC proliferation, it would be consistent with the > 2-fold increase in percent ECs by FACS in both *Mgp*^*f/f*^*;Ve*^*cre*^ and *Mgp*^*f/f*^*;Ad*^*cre*^ iBAT (Figure 8B). Minor changes in the expression of *Alk2* receptor, *Smad1*, and the inhibitory *Smad6* and *Smad7* supported activation of the endothelial BMP signaling, and were consistent with the BMP activation in the full iBAT from the *Mgp*^*f/f*^*;Ve*^*cre*^ and *Mgp*^*f/f*^*;Ad*^*cre*^ mice, respectively (Figure 7D). The vascular pattern in iBAT showed most spacing in the *Mgp*^*f/f*^*;Ve*^*cre*^ iBAT, as visualized by immunofluorescence for CD31 (Figure 8D). The area covered by vasculature was most prominent in the *Mgp*^*f/f*^*;Ad*^*cre*^ iBAT (Figure 8D), where the vessel diameters was the smallest (Figure 8D). The vascular patterning reflected brown versus white adipose differentiation and the size of the lipid droplets. Thus, in both the *Mgp*^*f/f*^*;Ve*^*cre*^ and *Mgp*^*f/f*^*;Ad*^*cre*^ iBAT, the ECs showed enhanced proliferation and expression of EC markers and BMP4, which has also been shown in MGP-depleted ECs in prior studies [34,49], suggesting that MGP from either ECs and adipose cells can affect the ECs. It suggests that MGP plays a direct role in EC biology.

Altogether, our results suggest that MGP is important in early specification of brown progenitor cells in the perivascular region, transition to maturity of especially CD142+ adipose cells, and regulation of adipose capillary growth (Supplemental Figure 11 for schematic model).

## 4 Discussion

BAT differs from WAT during pre- and postnatal development and remodeling in that it is fully developed prior to birth, as determined by tissue mass and *Ucp1* expression [8], which enables the pups to maintain body temperature. Postnatally, the BAT continues to grow, first due to proliferation until P14, and subsequently due to lipid accumulation [8]. In mouse WAT, a tentative hierarchy has been defined among the ASPCs during development [1,20]. In young mice, DPP4+ interstitial progenitor cells give rise to CD142+ cells Aregs and ICAM1+ preadipocytes. The latter are primed to differentiate into mature adipocytes [20], with possible interconversion between the two. The DPP4+ cells may provide a renewable source of preadipocytes, or alternatively, the ICAM1+ and CD142+ cells may mediate tissue turnover and remodeling unrelated to the DPP4+ cells. In our analysis of the BAT scRNA-seq data, the ASPCs have a similar composition as the progenitor populations identified in WAT, including ASCs (DPP4+, CD55+), pre-adipocytes (ICAM1+) and Aregs (CD142+), suggesting that the progression of differentiation in BAT at least in part parallels that of WAT.

In our study, we focused on the endothelial and adipose progenitor populations. We found that *Mgp* is expressed in a distinct sub-population of ECs, which might correspond to previous observations that MGP is expressed with specific timing during angiogenesis [44]. In addition, *Mgp* is highly expressed in the CD142+ adipose progenitor cells, which suggests a potential role for MGP in support of *Cd142*-expressing cell transitions. The results pointed to at least two separate roles in the brown adipogenic development, one related to ECs and the other to adipose progenitors. Because the phenotype of the global *Mgp*-KO mouse was very complex with mixed endothelial and adipose phenotypes, we chose to perform cell-specific *Mgp*-deletions using the *Ve-Cre* and *Adipoq-Cre* mice in attempt to distinguish effects in ECs versus adipose cells. Since MGP is a secreted protein, we presumed that the EC-specific deletion would also target the perivascular region directly surrounding the maturing adipocytes.

Our results suggested that the EC-derived MGP is necessary to ensure that the lineage specification is directed towards brown adipogenesis in the perivascular region. On the other hand, MGP secreted from the CD142+ cells helps bridge the transition of these progenitors to brown adipogenic maturity, thereby playing a role in the regulation of the number of cells required for BAT function and preventing excess cells to remain at earlier stages. The large size of the non-functional MGP-deficient iBAT may reflect such a disruption, where large numbers of immature cells flood the tissue. The RNA seq analysis (Supplemental Figure 7) showed that the wild-type iBAT had high expression of metabolic transcripts, which were down-regulated in the *Mgp*-KO iBAT. Thus, MGP seems to form a series of regulatory locks to direct BAT development. Sc-RNAseq information from other mouse and human BAT (data sets GSE161447 and E-MTAB-8564) show similar patterns of *Mgp* expression in subsets of ECs and adipose progenitors (data not shown).

BMP4 is known to cause commitment of mesenchymal stem/progenitor cells into adipocytes [24], which may occur perivascularly; BMP4 is a dominant BMP in the vascular endothelium [23,50] where it can also induce *Mgp* and establish a negative feedback regulation that involves BMP4 and MGP [31,51]. The whitening of the BAT after the endothelial *Mgp*-deletion suggest enhanced BMP4 activity as MGP was reduced in the perivascular region. The perivascular region is likely to serve as a target of whitening also in other contexts such as diabetes, which has been associated with enhanced BMP4 expression [52], potentially affecting the metabolic and thermogenic capacities of BAT. Indeed, endothelial *Bmp4* expression is highly responsive to hyperglycemia, hyperlipidemia, and inflammation [23,50], and is increased in obese humans where BMP4 correlates positively with adipocyte size [22]. Enhanced circulating levels of BMP4 from hepatic overexpression have been associated with reduced UCP1 levels, impaired lipolytic activation and increased lipids in BAT in mice [53]. Furthermore, adenoviral overexpression of BMP4 in adipose tissue enhances lipid droplet size but decreased expression of brown adipogenic markers [54]. Thus, BMP4 can affect both BAT and WAT and promote white adipogenic characteristics.

The importance of BMP regulation was also shown in our previous studies where adipocyte-specific deletion of *Noggin (Nog)*, a well-known BMP inhibitor that binds similar BMPs as MGP [21]. *Nog* deletion in mice was associated with age-related obesity in both genders unrelated to food intake, causing WAT hypertrophy and impaired function in BAT [55]. Noggin was also reduced in diet-induced obesity in inbred mice [55]. In the current data sets, expression of *Nog* was very low in the progenitor cells, supporting a role for Noggin in long-term regulation or age-related weight maintenance. Interestingly, no significant effect was detected after endothelial *Nog* knockout [55], supporting that MGP is a dominant inhibitor in the adipose endothelium.

The vascular effects of MGP is likely reinforced by its molecular characteristics. MGP is known to bind several other matrix proteins including fibronectin, HSP70, and is also part of matrix vesicles [[56], [57], [58]]. Thus, MGP may be highly effective in binding and inhibiting BMPs in precise locations such as the perivascular region or specialized matrix. It is less water soluble than Noggin, which might diffuse more freely and neutralize inappropriate BMP activity during adipose maintenance. Both the global and the cell-specific *Mgp* deletions expanded the EC population and the capillary network, with increased number of ECs, enhanced expression of EC markers, and enhanced BMP4 expression in the ECs from both mice with conditional *Mgp* deletions. There was even evidence of AV shunting in the iBAT with global *Mgp* deletion. It is consistent with previous studies where MGP was shown to be a potent vascular morphogen [33,35,43,44,49], and suggest that the ECs can be impacted by MGP deficiency in ECs as well as adipose cells, an advantage given the highly plastic nature of adipose tissue.

It is known that BMP signaling may need a specific temporal window to function correctly in developmental sequences or differentiation protocols to allow for correct activation of downstream signaling and cell specification [44,59,60]. The presence of BMPs and BMP antagonists might provide a more adaptable regulation of adipose tissue expansion and contraction, similar to previous observations in human white and beige adipogenesis [61]. The presence of BMP antagonists could also help mediate short pulses of BMP activity without large swings in BMP expression. In our case, MGP would be able to limit the exposure of ASPCs to excess BMP4 secreted by the endothelium.

We have previously shown that MGP binds and inhibits BMP7 [33], which has been strongly linked to brown adipogenesis. BMP7 promotes cultures of brown preadipocytes to undergo brown adipogenic differentiation, and when deleted in mice, causes a 50–70 % decrease in iBAT mass [25]. BMP7 delivered by minipumps also showed an increase in BAT volume in C57BL6/J mice [62]. Furthermore, administration of BMP7 in mice improved metabolic parameters and restored cellular glucose uptake with attenuated insulin signaling [63], and AAV-BMP7-mediated increases in BMP7 induced browning of WAT and activation of BAT [64]. Our results suggest that *Mgp* is expressed subsequently to *Bmp7*, which is expressed in the DPP4+ cells (Figure 4), thereby limiting BMP7 activity to the appropriate window of time during differentiation. The relationship of MGP to BMP8b and BMP6, which are closely related to BMP7, remains to be clarified. BMP8b is active in BAT but seems to be less involved in its development [28,29].

In summary, MGP is important in directing early brown adipogenic specification in perivascular progenitor cells, as well as in the transition to maturity of CD142+ adipose cells. Furthermore, both EC-derived and adipose cell-derived MGP can regulate the adipose capillary network.

## Funding

National Institutes of Health grant R01HL81397 (KIB).

National Institutes of Health grant R01HL158053 (KIB).

National Institutes of Health grant R01NS79353 (YY).

National Institutes of Health grant R01HL139675 (YY).

National Institutes of Health grant R01HL162643 (YY).

National Institutes of Health training grant T32HL007895 (XC).

National Institutes of Health grant K08HL168147 (XC).

UCLA Specialty Training and Advanced Research (STAR) fellowship program (XC).

## Author Contributions

Conception and design: LZ, XW, KIB

Collection and assembly of data: LZ, XQ, JJ, JM, LV

Data analysis and interpretation: LZ, XC, FM, YY, XW, KIB

Manuscript writing: LZ, XC, XW, KIB

Financial support: YY, KIB

Administrative support: KIB

## Declaration of competing interest

The authors declare that there are no conflicts of interest.

## Data Availability

Data will be made available on request.
