# Supplementary material for: Two-step regulation by matrix Gla protein in brown adipose cell differentiation
Source: Mol Metab. 2024 Jan 4;80:101870. doi: 10.1016/j.molmet.2024.101870 (PMC10832489; doi:10.1016/j.molmet.2024.101870)
Supplement: Multimedia component 1 [file mmc1.pdf]

Supplemental Table 1: KEY RESOURCES

REAGENT or RESOURCE	SOURCE	IDENTIFIER
**Antibodies - Immunofluorescence**		
CD31, goat polyclonal	R&D Systems	Cat# AF3628
ICAM1, mouse monoclonal	Proteintech	Cat# 60299-1-Ig
MGP, rabbit polyclonal	Abcam	Cat# ab86233
Perilipiin-1, rabbit polyclonal	Cell Signaling Technology	Cat# 9349S
**Antibodies - FACS**		
ICAM1 Alexa Fluor 647	Thermo Fisher	Cat#A15397
Mouse DPPIV/CD26 Alexa Fluor 594	R&D	Cat# FAB9541T-100UG
CD31 PE	Thermo Fisher	Cat#12-0311-82
CD140a/PDGFRA BV786	BD Biosciences	Cat# 740930
CD45 APC	Thermo Fisher	Cat# 17-0451-82
CD142 / F3, rabbit polyclonal	Sino Biological	Cat# 50413-R001
DPP4, mouse monoclonal	R&D Systems	Cat# AF954
PDGFRA, goat polyclonal	R&D Systems	Cat# AF1062
**Antibodies - Immunoblotting**		
FABP4 rabbit monoclonal	Cell Signaling Technology	Cat# 3544
Acetyl-CoA Carboxylase rabbit monoclonal		Cat# 3676
Fatty Acid Synthase rabbit monoclonal		Cat# 3180
Adiponectin rabbit monoclonal		Cat# 2789

PPARgamma rabbit monoclonal	Cell Signaling Technology	Cat# 2435
Phospho-HSL rabbit monoclonal		Cat# 4137
HSL rabbit monoclonal		Cat# 4107
SMAD1 rabbit monoclonal		Cat# 6944
Phospho-SMAD1/5 rabbit monoclonal		Cat# 9516
GAPDH rabbit monoclonal		Cat# 2118
SMAD4 rabbit monoclonal		Cat# 36535
SCD1 goat polyclonal	Santa Cruz Biotechnology	Cat# Sc14719
UCP1 rabbit UCP1 polyclonal	Abcam	Cat# ab10983
**Secondary antibodies and DAPI**		
4’,6-diamidino-2-phenylindole (DAPI)	Sigma-Aldrich	Cat# D9542
Chicken anti goat secondary antibodies, Alexa594	Invitrogen	Cat# A-21468
Chicken anti rabbit secondary antibodies, Alexa488	Invitrogen	Cat# A-21441
**Cell Lines, Medium, and Chemicals**		
Pre-BAT cells	Dr. Aldons J. Lusis, UCLA	N/A
BODIPY™ 493/503	Thermo Fisher	Cat# D3922
Oil Red O	Sigma	Cat# O0625
DMEM, high glucose	Gibco	Cat# 11965118
3-Isobutyl-1-methylxanthine	Sigma	Cat# I7018-100mg
3,3′,5-Triiodo-L-thyronine sodium salt	Sigma	Cat# T6397-250mg

Indomethacin	Sigma	Cat# I7378-5g
Dexamethasone	Sigma	Cat# D4902-100mg
Insulin from Bovine pancreas	Sigma	Cat# I5500-100mg
Rosiglitazone	Sigma	Cat# R2408-50mg
Antibiotic-Antimycotic (100x)	Gibco	Cat# 15240062
LY294002 (DMSO solution)	Abcam	Cat# ab146593
**Immunoblotting**		
Nitrocellulose Membrane	Invitrogen	Cat# 88018
NuPAGE™ Transfer Buffer (20X)		Cat# NP00061
NuPAGE™ MES SDS Running Buffer (20X)		Cat# NP000202
NuPAGE™ LDS Sample Buffer (4X)		Cat# NP0007
NuPAGE™ Sample Reducing Agent (10X)		Cat# NP0009
NuPAGE™ 10%, Bis-Tris, 1.0–1.5 mm, Mini Protein Gels		Cat# NP0301BOX
RIPA Lysis and Extraction Buffer	Thermo Scientific	Cat# 89901
PageRuler™ Prestained Protein Ladder, 10 to 180 kDa	Thermo Scientific	Cat# 26616
Phosphatase inhibitor	Roche	Cat# 04906837001
Protease inhibitor	Roche	Cat# 11836153001
**Other chemicals**		
Antigen Unmasking Solution	Vector Laboratories	Cat# H-3300-250
Erythrocyte Lysis Buffer	Qiagen	Cat# 79217
2-Mercaptoethanol	Gibco	Cat# 21985023
**Virus Strains and Transfection Reagents**		

MGP shRNA Lentiviral particles	Santa Cruz Biotechnology	SC-44627
Control ShRNA Lentiviral particles	Santa Cruz Biotechnology	Sc108080
Polybrene	Santa Cruz Biotechnology	Cat# SC-134220
Puromycin	Sigma-Aldrich	Cat# P9620
**Essential Commercial Assays**		
Corning™ Sterile Cell Strainers 40μm	Corning	Cat# 431750
RNeasy Mini Kit	Qiagen	Cat# 74106
High-Capacity cDNA Reverse Transcription Kit	Thermo Fisher	Cat# 4374967
TaqMan™ Universal Master Mix II, With UNG	Thermo Fisher	Cat# 4440039
HiPerFect Transfection Reagent	Qiagen	Cat# 301704
FluoSpheres™ Polystyrene Microspheres, 15 μm, green fluorescent (450/480)	Invitrogen	Cat# 21010
**Deposited Data**		
WT/KO BAT	GEO	GSE233274
**Software and Algorithms**		
GraphPad Prism	GraphPad Software Inc.	https://www.graphpad.com/scientific-software/prism/
Cell ranger v3.1.0	10x Genomics	https://support.10xgenomics.com
Seurat v3.1.2		https://satijalab.org/seurat/

g:Profiler		https://biit.cs.ut.ee/gprofiler/gost
