# Supplementary material for: Two-step regulation by matrix Gla protein in brown adipose cell differentiation
Source: Mol Metab. 2024 Jan 4;80:101870. doi: 10.1016/j.molmet.2024.101870 (PMC10832489; doi:10.1016/j.molmet.2024.101870)
Supplement: Multimedia component 2 [file mmc2.pdf]

Supplemental Table 2. Primers for qRT-PCR and genotyping

Primer	Oligonucleotides/ Cat#	Experiment
GAPDH	Mm99999915_g1	RT-qPCR
BMP4	Mm00432087_m1	RT-qPCR
BMP7	Mm00432102_m1	RT-qPCR
Mgp	Mm01615497_m1	RT-qPCR
Prdm16	Mm00712556_m1	RT-qPCR
Plin1	Mm00558672_m1	RT-qPCR
Pparg	Mm00440940_m1	RT-qPCR
F3	Mm00438855_m1	RT-qPCR
Pdgfra	Mm00440701_m1	RT-qPCR
Dpp4	Mm00494552_m1	RT-qPCR
Pgc1a	Mm0128835_m1	RT-qPCR
Adipoq	Mm00456425_m1	RT-qPCR
Cd34	Mm00476712_m1	RT-qPCR
Kdr	Mm01222421_m1	RT-qPCR
Cdh5	Mm00486938_m1	RT-qPCR
Flt1	Mm00438980_m1	RT-qPCR
Pecam1	Mm00476712_m1	RT-qPCR
Ucp3	Mm01163394_m1	RT-qPCR
Tfam	Mm00447485_m1	RT-qPCR
Nrf1	Mm01135606_m1	RT-qPCR
Bmp4	Mm00432087_m1	RT-qPCR

Bmp2	Mm01340178_m1	RT-qPCR
Bmp6	Mm01332882_m1	RT-qPCR
Bmp3b/Gdf10	Mm01220860_m1	RT-qPCR
Gdf5	Mm00433564_m1	RT-qPCR
Bmp7	Mm00432102_m1	RT-qPCR
Bmp8a	Mm00432109_m1	RT-qPCR
Bmp8b	Mm004321115_m1	RT-qPCR
Grem1	Mm00488615_s1	RT-qPCR
Fst	Mm00514982_m1	RT-qPCR
Bambi	Mm03024088_g1	RT-qPCR
Bmpr2	Mm00432134_m1	RT-qPCR
Alk6/Bmpr1b	Mm03023971_m1	RT-qPCR
Alk3/Bmpr1b	Mm00477650_m1	RT-qPCR
Alk2/Acvr1	Mm01331069_m1	RT-qPCR
Alk1/Acvrl1	Mm00437432_m1	RT-qPCR
Smad7	Mm00484742_m1	RT-qPCR
Smad6	Mm00484738_m1	RT-qPCR
Smad4	Mm03023996_m1	RT-qPCR
Smad1	Mm00484723_m1	RT-qPCR
Mgp_WT-F	GCCACAATTTCTGCATCCTGC	Genotyping
Mgp_WT-R	CGGGAAAGATGAGGAAGAAGGG	Genotyping
MGP-mutant-F	TGCCTGAAGTAGCGGTTGTA	Genotyping
MGP-mutant-R	TGAATGAACTGCAGGACGAGG	Genotyping

Adipoq-tg-F	ACGGACAGAAGCATTTTCCA	Genotyping
Adipoq-tg-R	GGATGTGCCATGTGAGTCTG	Genotyping
Mgp^{flox}-F	CTTAGAAACTGCTGGGTCACGTAGC	Genotyping
Mgpflox-R	CTTATTTGTCACACGCTGTCACTGTC	Genotyping
Ve-tg-F	AGGCAGCTCACAAAGGAA CAA T	Genotyping
VE-tg-R	TCG TTG CAT CGA CCG GTA A	Genotyping
$Adipoq^{Ert2}$-tg-F	GAG TCT GCC TTT CCC ATG AC	Genotyping
$Adipoq^{Ert2}$-tg-R	TCC CTC ACA TCC TCA GGT TC	Genotyping
mTmG-common	CTT TAA GCC TGC CCA GAA	Genotyping
mTmG-mutant-F	TAG AGC TTG CGG AAC CCT TC	Genotyping
mTmG-Wild-type-F	AGG GAG CTG CAG TGG AGT AG	Genotyping
