# Supplementary material for: Two-step regulation by matrix Gla protein in brown adipose cell differentiation
Source: Mol Metab. 2024 Jan 4;80:101870. doi: 10.1016/j.molmet.2024.101870 (PMC10832489; doi:10.1016/j.molmet.2024.101870)
Supplement: Multimedia component 3 [file mmc3.pdf]

SUPPLEMENTAL FIGURES

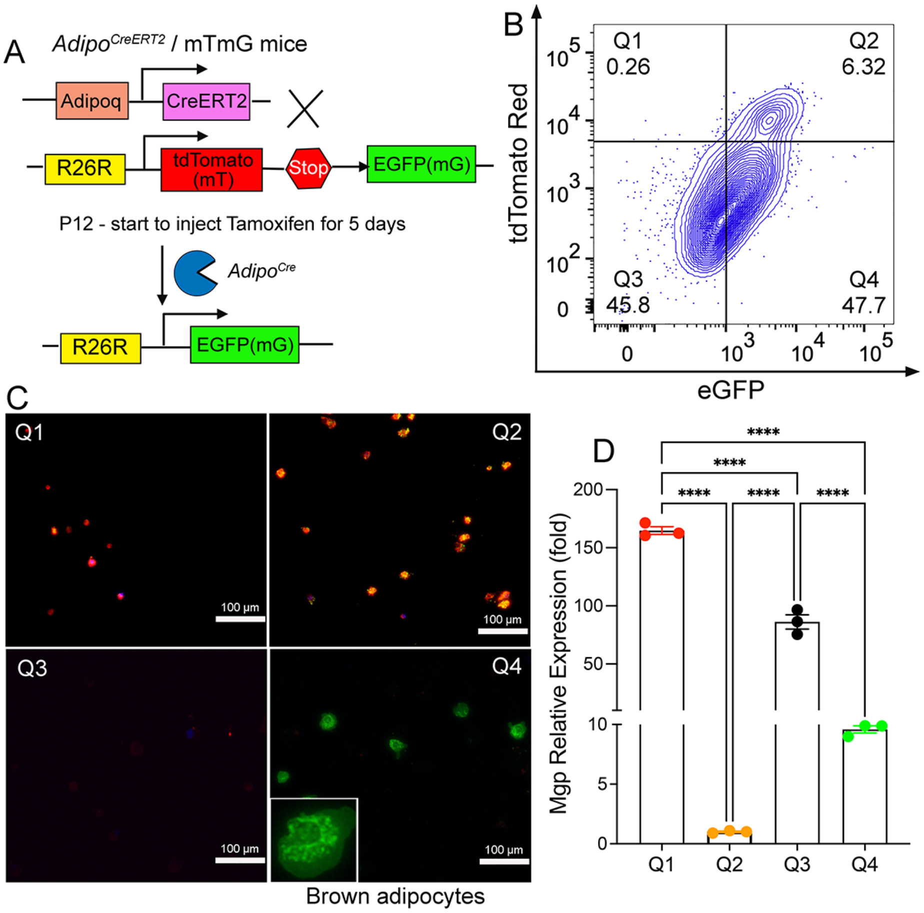

Supplemental Figure 1

Determination of MGP expression in mature brown adipocytes.

(**A**) Schematic of mouse breeding to generate mice with selective expression of eGFP in cells expressing Adiponectin. The mice were injected with Tamoxifen on day 1, and continued for total of 5 days. As the adipocytes matured, the Adipo-Cre was activated and removed the red tdTomato (mT) leaving only the green eGFP (mG). After about 2 weeks, the cells were sorted by FACS.
(**B**) FACS analysis of iBAT cells isolated from *Adipo^{CreERT2};mTmG* mice (n=5 mice, 4 weeks of age), based on tdTomato Red and eGFP expression. Q4 showed only eGFP expression and represented mature brown adipocytes.
(**C**) Visualization of cells expressing fluorescent tdTomato Red (Q1), combine tdTomato Red and eGFP (Q2), and eGFP (Q4). Inset, enlargement of eGFP. Bars, 100 µm.
(**D**) Expression of Mgp in Q1-4 sorted cells, as determined by qPCR (n=3).
Data are shown as mean+SEM; One way ANOVA, **** p<0.0001.

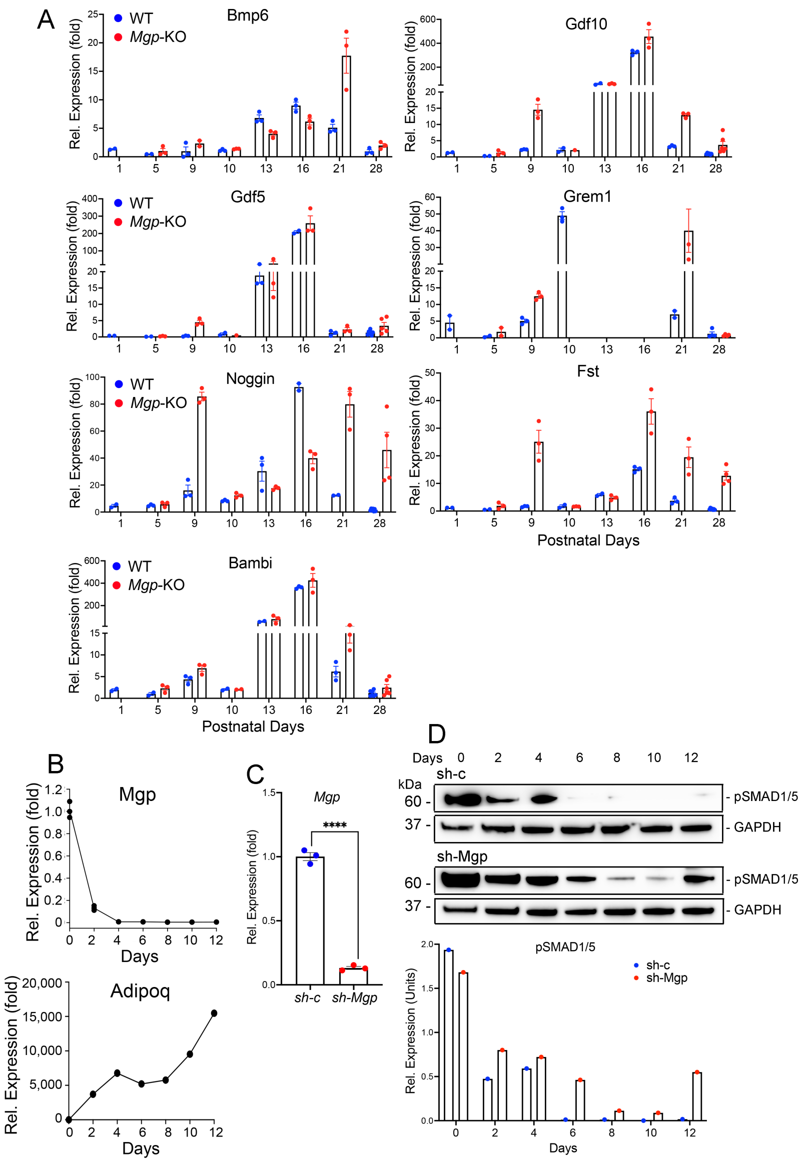

Supplemental Figure 2

BMP agonists, inhibitors, and SMAD signaling in iBAT and brown adipose cells.

(**A**) Time course of expression of the agonists *Bmp6, Gdf10 (Bmp3b), Gdf5 (Cdmp1), and the inhibitors Grem1, Nog, Fst and Bambi* in iBAT from wild type (WT) and global *Mgp*-knockout (KO) mice between postnatal day (p)1-28, as determined by qPCR. Expression is calculated as fold change compared to p1 (n=3)

(**B**) Expression of *Mgp* and *Adipoq*, by qPCR in pre-BAT cells cultured for up to 12 days, as determined by qPCR. Expression is calculated as fold change compared to day 0 (representative of 3 replicate experiments).

(**C**) *Mgp* shRNA and control shRNA were used to transfect pre-BAT cells using 5 µg/ml Polybrene and cultured for up to 12 days. Mgp expression was reduced to 10-15% of normal levels by the *Mgp* shRNA transfection.

(**D**) MGP deficiency in pre-BAT cells prolongs SMAD signaling. Immunoblotting of activated pSMAD1/5 after transfection of control shRNA (top) or *Mgp* shRNA (bottom), quantified by densitometry (representative of 3 replicate experiments).

Data from qPCR (panel C) are shown as mean$\pm$SEM; unpaired two-tailed Student's *t* test, **** $p<0.0001$.

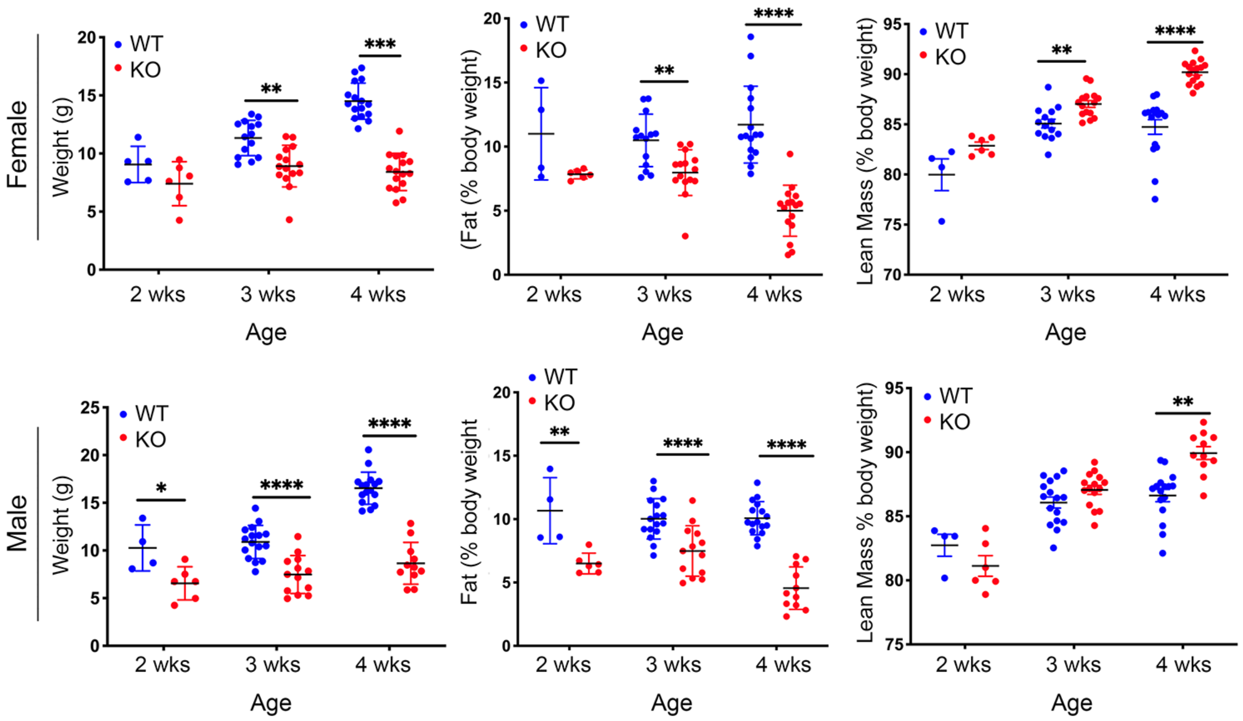

Supplemental Figure 3

Comparison of body composition in wild type with global Mgp-knockout mice

Body weight, percent fat and percent lean mass in female and male wild-type (WT) and *Mgp*-knockout (KO) mice, from 4 weeks of age (data from n=5-12 mice per group).
Data are shown as mean+SEM; One way ANOVA, * p<0.05, ** p<0.001, *** p<0.001, **** p<0.0001.

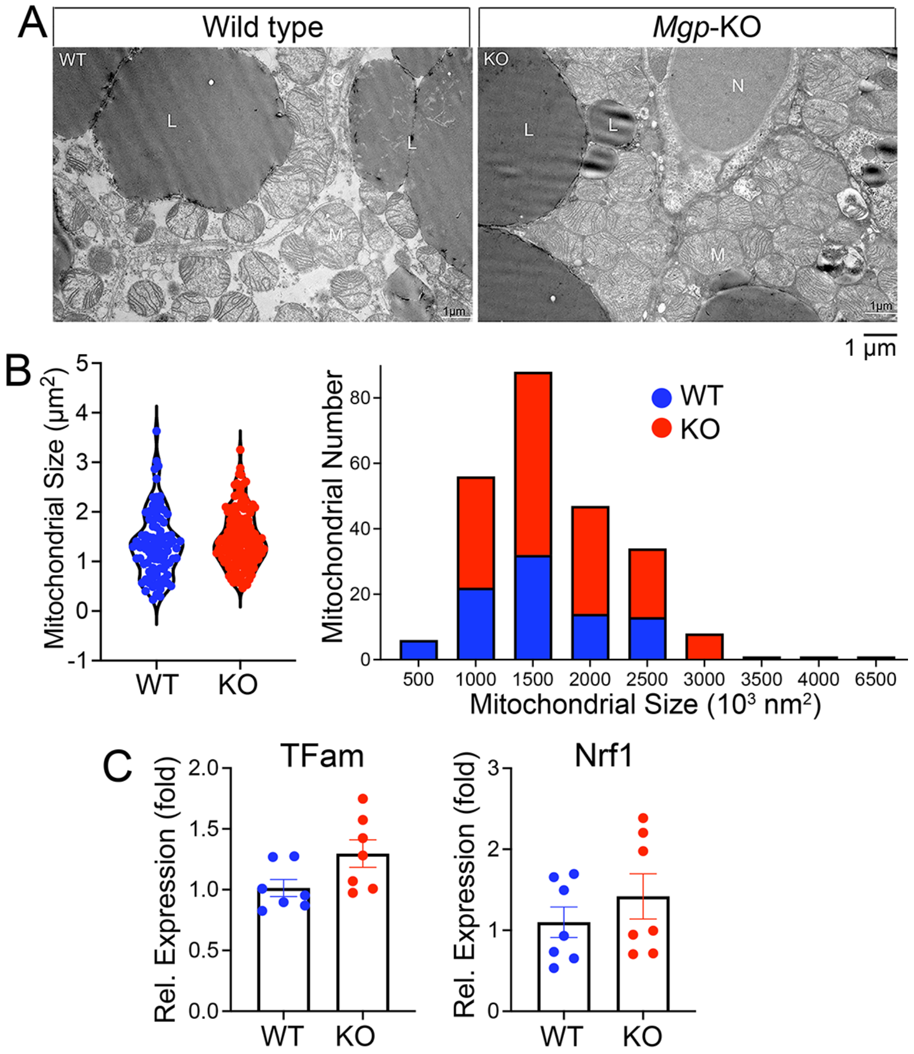

Supplemental Figure 4

Global Mgp-deletion enhances the number of mitochondria in brown adipocytes

(**A**) Electron microscopy of interscapular (i)BAT demonstrating an excess of mitochondria in *Mgp*-KO mice as compared to wild type (WT) mice. Bars, 1 um.
(**B**) Mitochondrial size (left) and mitochondrial number (right) in WT and *Mgp*-KO iBAT.
(**C**) Expression of the thermogenesis-related genes *TFam* and *Nrf1* in WT and *Mgp*-KO iBAT, as determined by qPCR (n=7 mice per group).
Data from qPCR are shown as mean±SEM. Unpaired two-tailed Student's *t* test.

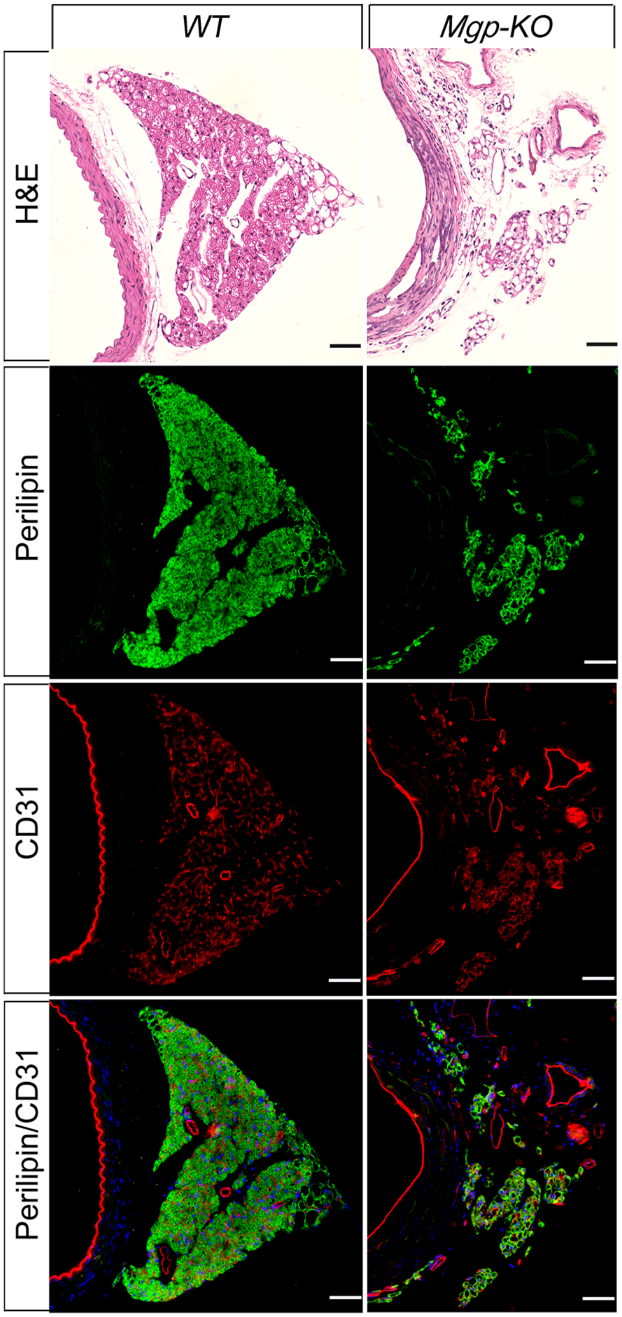

Supplemental Figure 5

Global MGP deficiency disrupts aortic perivascular adipose tissue (PVAT).

(Top panels) H&E staining of PVAT from mid-dorsal aorta.
(Bottom panels) Immunofluorescence for Perilipin (green), CD31 (red), and merged Perilipin/CD31 in PVAT from wild type (WT) and *Mgp*-knockout (KO).
Bars, 25 µm.

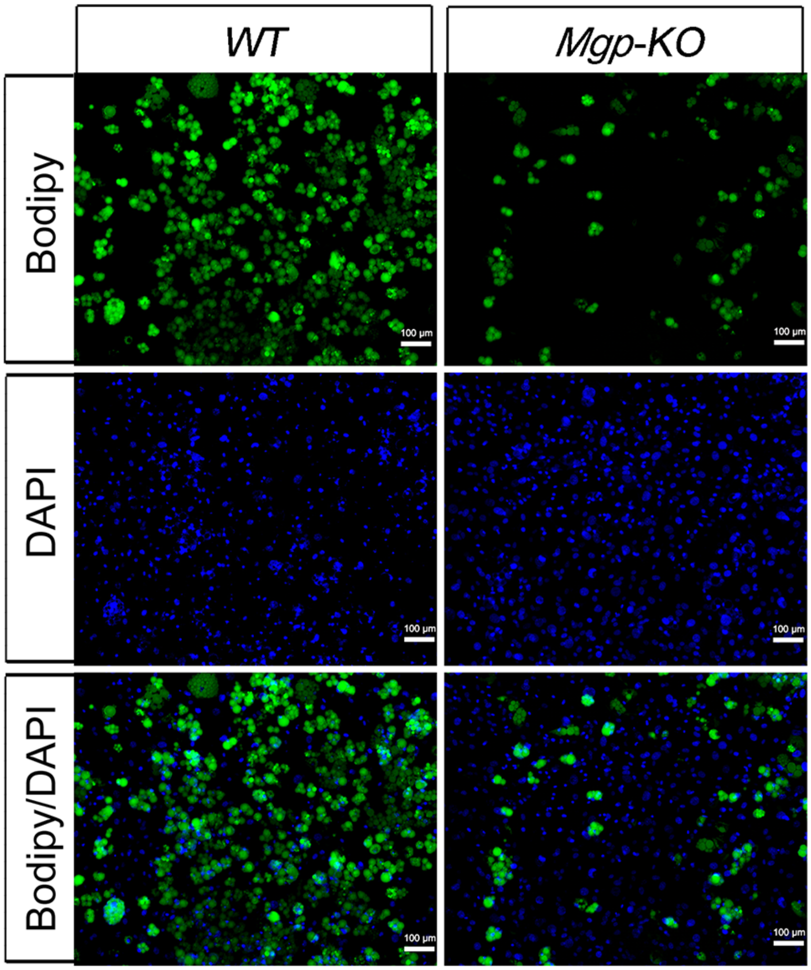

Supplemental Figure 6

MGP deficiency disrupts adipogenesis in cultured PDGFRA+DPP4+ cells from BAT but does not reduce the cell numbers.

PDGFRA+DPP4+ cells were isolated from wild type (WT) and *Mgp*-knockout (KO) iBAT and cultured in adipogenic conditions for 12 days.
(Top panels) Staining for lipid droplets with BODIPY in PDGFRA+CD142+ cells from WT and *Mgp*-KO iBAT.
(Middle panels) Nuclear staining with DAPI.
(Bottom panels) Merged BODIPY/DAPI stainings.
Bars, 50 μm.

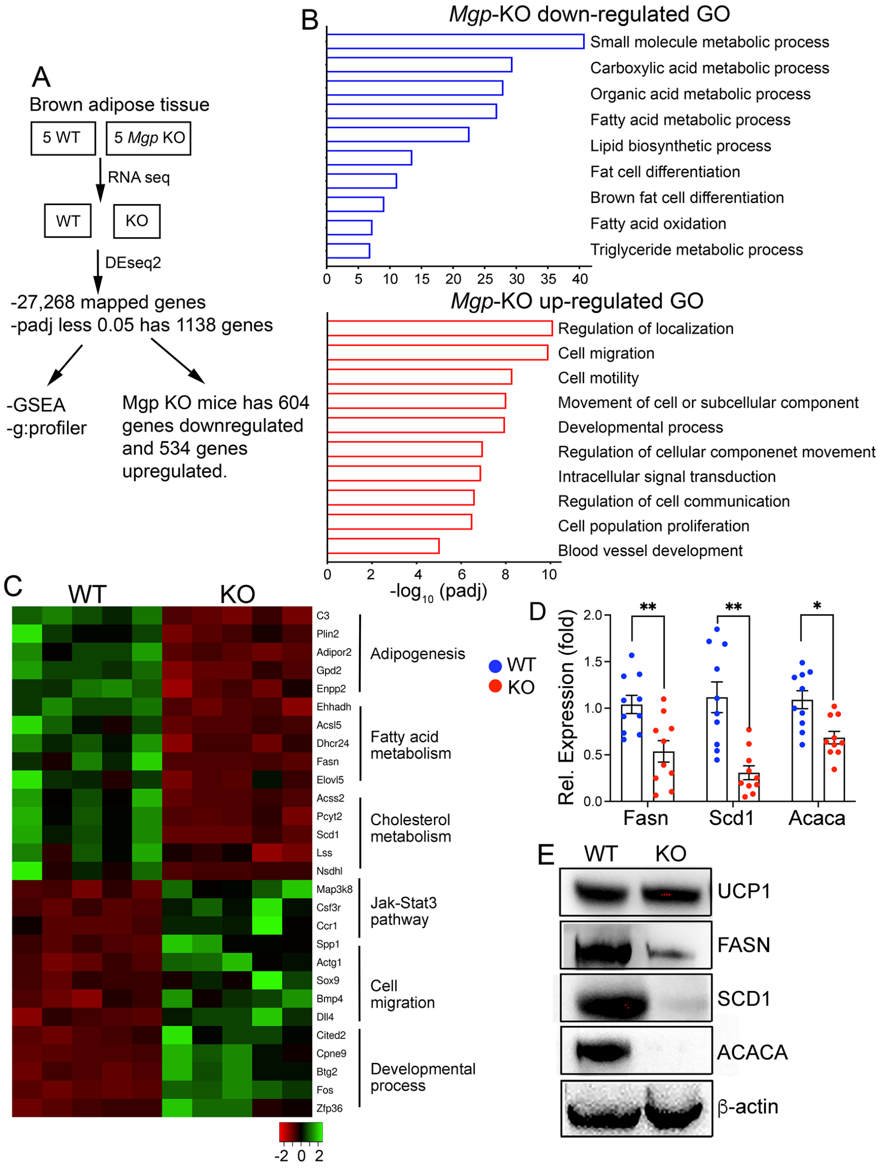

Supplemental Figure 7

RNA-seq analysis shows that MGP-deficiency promotes stem cell characteristics

(**A**) Schematic diagram of RNA-seq analysis strategy.
(**B**) Gene ontology (GO) analysis of down-regulated and up-regulated genes in iBAT from *Mgp*-knockout (KO) mice compared to wild-type (WT) mice (n=5 mice per group).
(**C**) Gene expression heat map of DEGs in iBAT from WT and KO mice.
(**D**) Expression of *Fasn*, *Scd1*, and *Acaca*, related to lipid biosynthesis, in iBAT from WT and KO mice, as determined by qPCR (n=10 mice). Data are shown as mean$\pm$SEM; unpaired two-tailed Student's *t* test, * $p<0.05$, ** $p<0.01$.
(**E**) Expression of UCP1, FASN, SCD1 and ACACA in iBAT from WT and KO, as determined by immunoblotting using iBAT from the same mice as for qPCR (n=6 mice per group). Beta-actin was used as loading control.

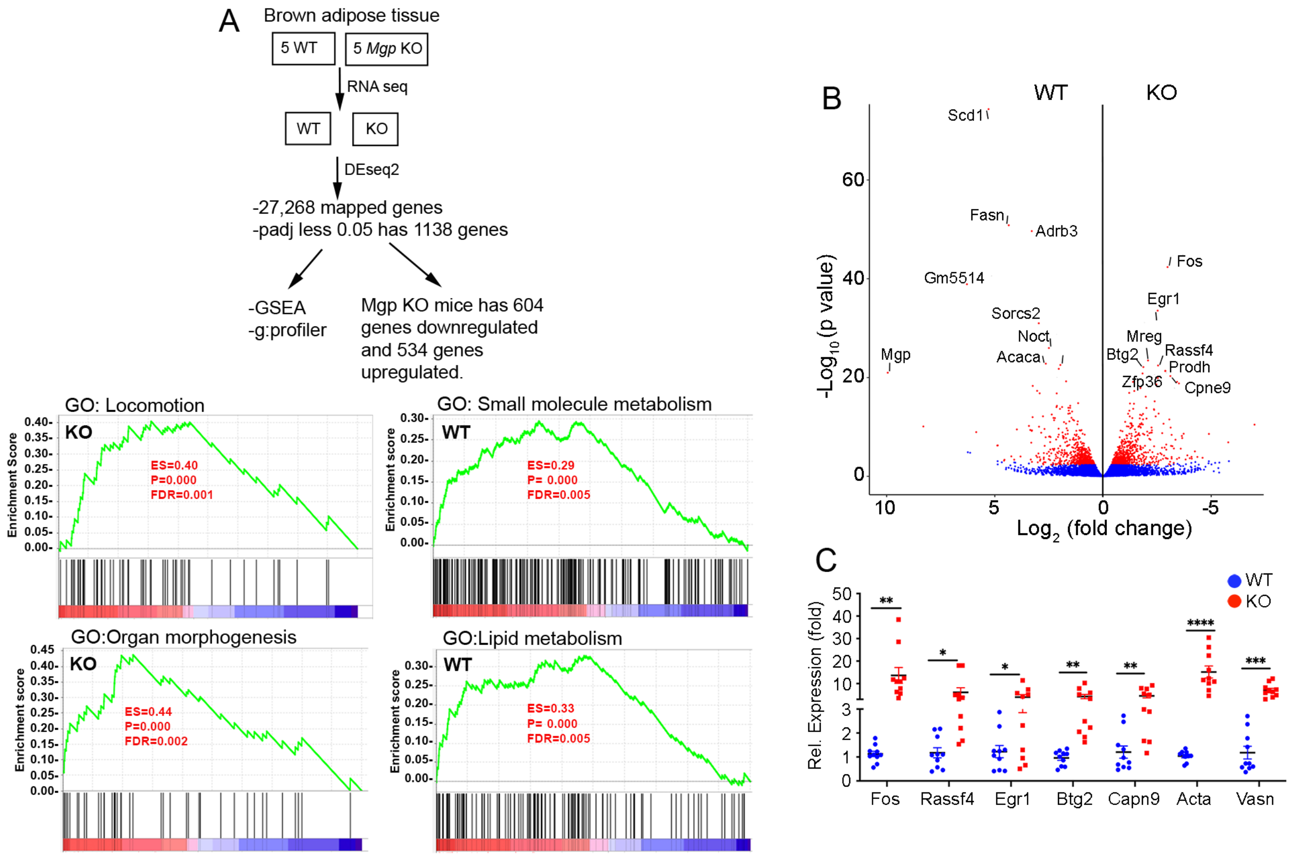

Supplemental Figure 8

Gene set enrichment analysis (GSEA) in iBAT from wild-type and Mgp-KO mice

(**A**) Schematic diagram of RNA-seq analysis strategy (top). GSEA analysis of 604 down-regulated and 534 up-regulated genes in iBAT from *Mgp*-knockout (KO) mice and wild-type (WT) mice (n=5 mice per group).
(**B**) *Global Mgp-deletion reduces gene expression related to lipid biosynthesis:* Volcano plot analysis of RNA-seq data reveals that global *Mgp*-deletion significantly reduces lipid metabolism. Lipid biosynthesis genes such as *Scd1*, *Acaca*, and *Fasn*, are all expressed in wild type (WT) mice, whereas stem cell markers such as *Fos*, *Egr1*, *Btg2*, *Zfp36*, show enhanced expression in the *Mgp*-KO iBAT.
(**C**) Expression of *Fos*, *Rassf4*, *Egr1*, *Btg2*, *Capn9*, *Acta1*, and *Vasn* was enhanced in the *Mgp*-KO iBAT compared to WT, as determined by qPCR (n=10 mice per group).
Data from qPCR are shown as mean±SEM. Unpaired two-tailed Student's *t* test, * p<0.05, ** p<0.01, *** p<0.001, **** p<0.0001.

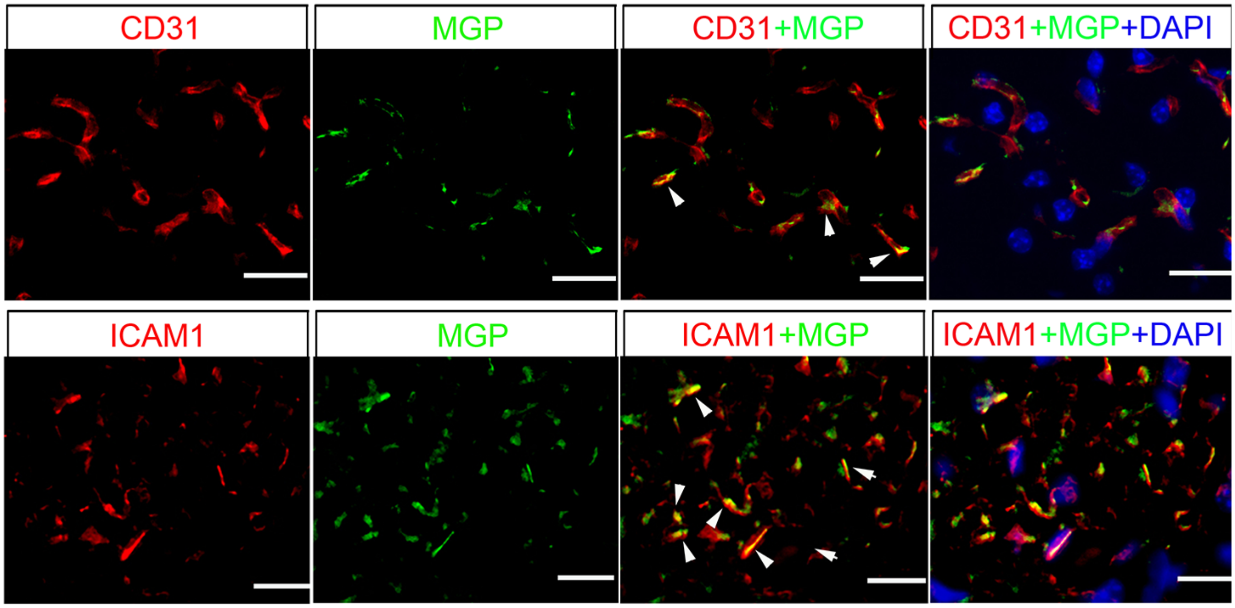

Supplemental Figure 9

Co-localization of MGP with CD31 and ICAM1 in wild-type iBAT

Immunofluorescence for CD31 (red) and MGP (green) (top panels), and ICAM1 (red) and MGP (green) (bottom panels) in iBAT from wild-type mice at 4 weeks of age. DAPI (blue) was used to visualize nuclei. Bars, 25 µm. White arrowheads indicate CD31 and MGP double-positive cells (top panel), or ICAM1 and MGP double-positive cells (bottom panel).

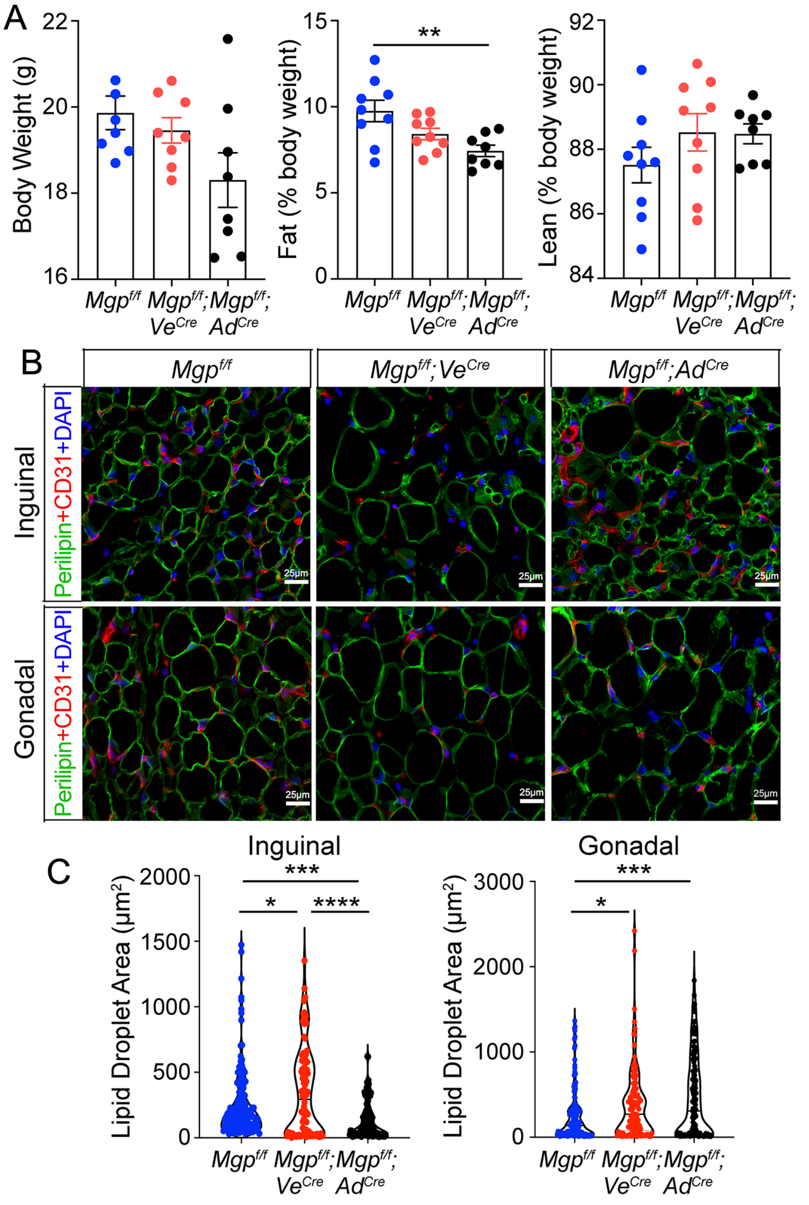

Supplemental Figure 10

Characterization of white adipose tissue (WAT) in mice with conditional Mgp deletions

(**A**) Body weight, percent fat and percent lean mass in *Mgp*$^{f/f}$ mice, *Mgp*$^{f/f}$ *: Ve*^{Cre} mice, and *Mgp*$^{f/f}$ *: Ad*^{Cre} mice, at 4 weeks of age (data from n=8-9 mice per group).
(**B**) Immunofluorescence for Perilipin (green) and CD31 (red) in inguinal and gonadal WAT in *Mgp*$^{f/f}$ mice, *Mgp*$^{f/f}$ *: Ve*^{Cre} mice, and *Mgp*$^{f/f}$ *: Ad*^{Cre} mice. DAPI was used to visualize the nuclei. Bars, 25 μm (representative of 3 replicates).
(**C**) Area of lipid droplets in inguinal and gonadal WAT from *Mgp*$^{f/f}$ mice, *Mgp*$^{f/f}$ *: Ve*^{Cre} mice, and *Mgp*$^{f/f}$ *: Ad*^{Cre} mice, as determined by ImageJ after staining for Perilipin (data from n=3 mice per group).
Data are shown as mean+SEM; One way ANOVA, * $p<0.05$, ** $p<0.001$***, $p<0.001$, **** $p<0.0001$.

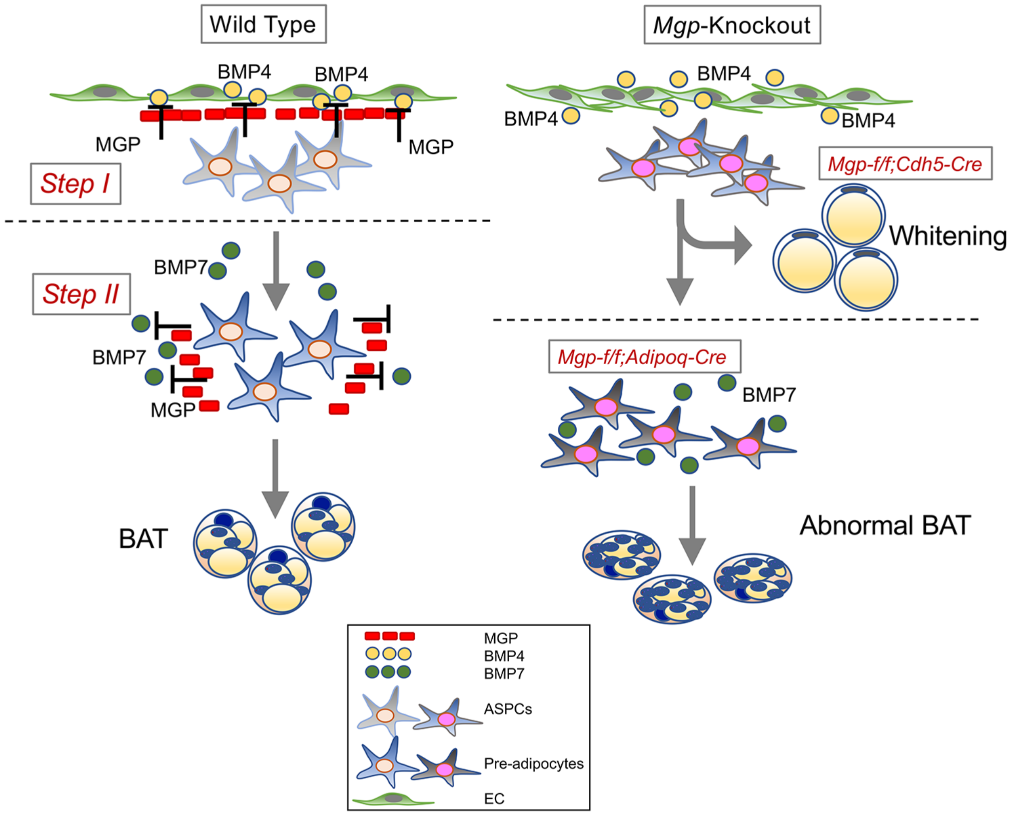

Supplemental Figure 11

Working model of matrix Gla protein (MGP)-regulated steps in brown adipogenesis

(Left panel) MGP limits endothelial BMP4 in the induction of white adipogenesis or "whitening" in the perivascular area (top). MGP derived from CD142+ cells regulate BMP7 from DPP4 cells in the bridging of the CD142+ cells to mature brown adipocytes (bottom).
(Right panel) *Mgp* gene deletion in the endothelial cells causes endothelial overgrowth and white adipogenic induction in adipose progenitor cells (top). *Mgp* gene deletion in the adipose cells causes excess lipolytic activity (bottom).
